# Glucagon-like peptide-1 receptor agonists as add-on therapy to insulin for type 1 diabetes mellitus: a systematic review and meta-analysis

**DOI:** 10.1007/s42000-025-00704-9

**Published:** 2025-08-04

**Authors:** Eleni Rebelos, Ioanna A. Anastasiou, Nikolaos Tentolouris, Thomas Karagiannis, Apostolos Tsapas, Ele Ferrannini, Aris Liakos

**Affiliations:** 1 First Department of Propaedeutic Internal Medicine and Diabetes Center, School of Medicine, National and Kapodistrian University of Athens, Laiko General Hospital, Athens, Greece; 2 Department of Clinical and Experimental Medicine, University of Pisa, 56126 Pisa, Italy; 3 Clinical Research and Evidence-Based Medicine Unit, Second Medical Department, Aristotle University of Thessaloniki, Thessaloniki, Greece; 4 Diabetes Centre, Second Medical Department, Aristotle University of Thessaloniki, Konstantinoupoleos 49, 54642 Thessaloniki, Greece; 5 Institute of Clinical Physiology, CNR, Pisa, Italy

**Keywords:** Type 1 diabetes, GLP-1 receptor agonist, HbA_1c_, Weight loss

## Abstract

**Aims:**

We conducted a systematic review to compare the efficacy and safety of glucagon-like peptide 1 receptor agonists (GLP-1 RAs) versus placebo in addition to insulin treatment in patients with type 1 diabetes (T1D).

**Methods:**

We searched the PubMed and Cochrane databases up to November, 2023. Randomized controlled trials (RCTs) with a duration of ≥ 12 weeks were deemed eligible for the primary outcome (change in HbA_1c_) and change in body weight. RCTs with a duration of ≥ 4 weeks were deemed eligible for the incidence of severe hypoglycemia, time-in-range (TIR), total insulin dose (TID), change in C-peptide, and glucagon levels. We calculated mean differences (MDs) for continuous outcomes and odds ratios (ORs) for severe hypoglycemia, alongside 95% CIs using an inverse-variance weighted random-effects model.

**Results:**

A total of 25 RCTs were eligible. Compared with placebo, use of GLP-1RA was associated with a reduction in HbA_1c_ (MD -0.23%, 95% CI -0.30, -0.17), body weight (MD -3.93 kg, 95% CI -4.29, -3.56), and TID (MD -5.74 U/day, 95% CI -7.30, -4.19). Addition of GLP-1RAs did not significantly affect time-in-range. Based on data from 13 studies, the odds for severe hypoglycemia were similar between patients receiving GLP1-RA and placebo. Qualitative assessment of individual trials showed that addition of GLP-1RA does not prevent progressive C-peptide loss and that patients receiving a GLP-1RAs do not have inadequate glucagon secretion in response to hypoglycemia.

**Conclusion:**

The addition of GLP-1RA on top of insulin therapy in people with T1D results in a modest reduction in HbA_1c_, body weight, and TID without increasing the risk for severe hypoglycemia.

**Supplementary Information:**

The online version contains supplementary material available at 10.1007/s42000-025-00704-9.

## Introduction

Type 1 diabetes (T1D) is characterized by autoimmune-mediated destruction of the pancreatic β-cells [1]. It has been reported that typically, by the time of diagnosis, ~ 80–90% of β-cells has been lost [2, 3]. B-cell destruction and loss of function continue over the years [4]; *however,* preserved C-peptide responses have been described even after long-standing T1D [5, 6]. Thus, early recognition of the disease and timely addition of disease-modifying interventions are of utmost importance to preserve β-cell function. Along with β-cell destruction, another key pathophysiologic feature of T1D is dysregulated glucagon secretion with deficient glucagon response to hypoglycemia and exaggerated glucagon release following a meal. This dysregulated pattern of glucagon secretion has been attributed to lack of intra-islet insulin secretion, although the mechanisms have not as yet been completely elucidated [7].

While the mainstay of T1D treatment is intensive insulin treatment either with multiple daily injections or continuous subcutaneous insulin infusion, efforts are underway to identify treatments that may preserve β-cell mass and function [8]. The relatively new antidiabetic drug class glucagon-like peptide 1 receptor agonists (GLP-1 RAs) stimulate β-cells to secrete more insulin without causing hypoglycemia. Moreover, GLP-1 suppresses glucagon secretion [9]. These drugs have been extensively used in patients with type 2 diabetes (T2D) in whom they lower HbA_1c_, body weight, and albuminuria [10]. Moreover, recent randomized controlled cardiovascular outcome trials (CVOTs) have shown that treatment with GLP-1RA reduces the risk of CV disease in high-risk subjects with T2D [10]. In recent years, whether addition of GLP-1RA in the treatment of patients with T1D also leads to beneficial outcomes in T1D and whether addition of these agents could preserve β-cell function has received a great deal of attention. A previous meta-analysis, including 11 randomized controlled trials (RCTs) that used liraglutide, exenatide, or albiglutide has shown that adjunctive therapy with GLP-1RA in T1D leads to improvement in HbA_1c_, weight loss, and decrease in total daily insulin dose (TID) [11]. These findings were later confirmed in a systematic review and meta-analysis of randomized controlled trials that included a larger number of RCTs [12]. Nevertheless, these systematic reviews did not include more recent RCTs using semaglutide or dulaglutide, which were not available at that time.

The aim of the present systematic review and meta-analysis was to expand examination of the effects of GLP-1RA in addition to insulin treatment in patients with T1D with regard to glycemic control, body weight, TID, time-in-range (TIR), and incidence of severe hypoglycemia. Additionally, for the first time, to the best of our knowledge, we aimed to evaluate whether addition of GLP-1RA on top of insulin treatment leads to preservation of C-peptide levels and to assess its effects on glucagon release.

## Materials and methods

We report our methods and results according to the Preferred Reporting Items for Systematic reviews and Meta-Analyses (PRISMA) 2020 statement [13]. The protocol of this systematic review and meta-analysis has been registered in PROSPERO (registration no. CRD42024513673).

### Search strategy

We systematically searched PubMed, and Cochrane Central Register of Controlled Trials from database inception up to 2 November, 2023. The detailed search strategy is shown in Supplementary Material. We additionally scanned international clinical trial registries (clinicaltrials.gov) EASD and ADA conference abstracts of the last 5 years.

### Study selection and data extraction

RCTs assessing the addition of any GLP-1RA currently in use (at any dose) compared to placebo on top of insulin in patients with T1D were eligible. Eligible studies were published in the English language. RCTs studying albiglutide were excluded as this drug is currently not in use. The primary outcome of interest was change in HbA_1c_ (%). Secondary outcomes were change in body weight, change in TIR, change in total insulin dose (TID), change in C-peptide levels, change in glucagon levels, and change in the incidence of severe hypoglycemia (defined as need for assistance from another person). RCTs with a duration of at least 12 weeks were deemed eligible for the change in HbA_1c_ and in body weight, whereas for all other outcomes a minimum duration of 4 weeks of intervention was required. We also included cross-over trials, which were handled as if they were parallel group trials [14]. A pair of reviewers (ER and IA) independently screened titles and abstracts, reviewed full texts of potentially eligible records, and extracted data from the included trials. Any disagreements were resolved by consensus. Multiple reports of the same study were identified and collated by one reviewer (ER) and double-checked by a second reviewer (IA). For each eligible study, we extracted data for study and participants’ baseline characteristics, as well as outcomes of interest using pilot-tested forms. When data were given in graphs, we used Plotdigitizer to extract the data of interest (https://plotdigitizer.com/). Missing measures of dispersion were imputed by previously published methodology [14] or by previous similar studies. These calculations are described in detail in Supplementary Material. For multiple-arm data, corrected standard errors for generic inverse variance (GIV) method analyses were calculated as previously described [15] (Supplementary Material).

### Risk of bias assessment

Two reviewers (ER and IA) independently assessed the risk of bias for the primary outcome using the revised Cochrane Collaboration Risk of Bias tool RoB2 for RCTs [16]. Studies that were not included in the assessment of the primary outcome were instead evaluated for the secondary outcome (total insulin dose). In brief, the risk of bias for each RCT was judged to be low if all domains were at low risk of bias and high if at least one domain was at a high risk of bias, while in all other cases RCTs were judged to have some concerns. Any discrepancies were resolved by consensus with a third reviewer (AL).

### Data synthesis

For continuous outcomes (change from baseline in HbA_1c_, body weight, TIR, and total insulin dose) we calculated weighted mean differences (MD) along with 95% confidence intervals using the generic inverse variance random-effects model. For dichotomous outcomes, random effects odds ratios and 95% CI were calculated by applying a constant continuity correction of 0.5 excluding zero total event trials. Statistical heterogeneity among studies was assessed with the *I*^*2*^ statistic, considering values greater than 50% as indicative of substantial heterogeneity [17]. A pre-specified subgroup analysis based on baseline C-peptide levels was also performed for the primary outcome. We explored presence of publication bias for the primary outcome by visually inspecting the funnel plot for asymmetry. All analyses were implemented in RevMan (version 5.4.1). Due to the large heterogeneity in the secretory stimuli and methods used to assess plasma C-peptide and glucagon levels in response to GLP-1RA vs placebo, it was not possible to perform meta-analyses for these secondary outcomes. For these outcomes, individual study results are described qualitatively.

## Results

### Search results and study characteristics

**Fig. 1:**
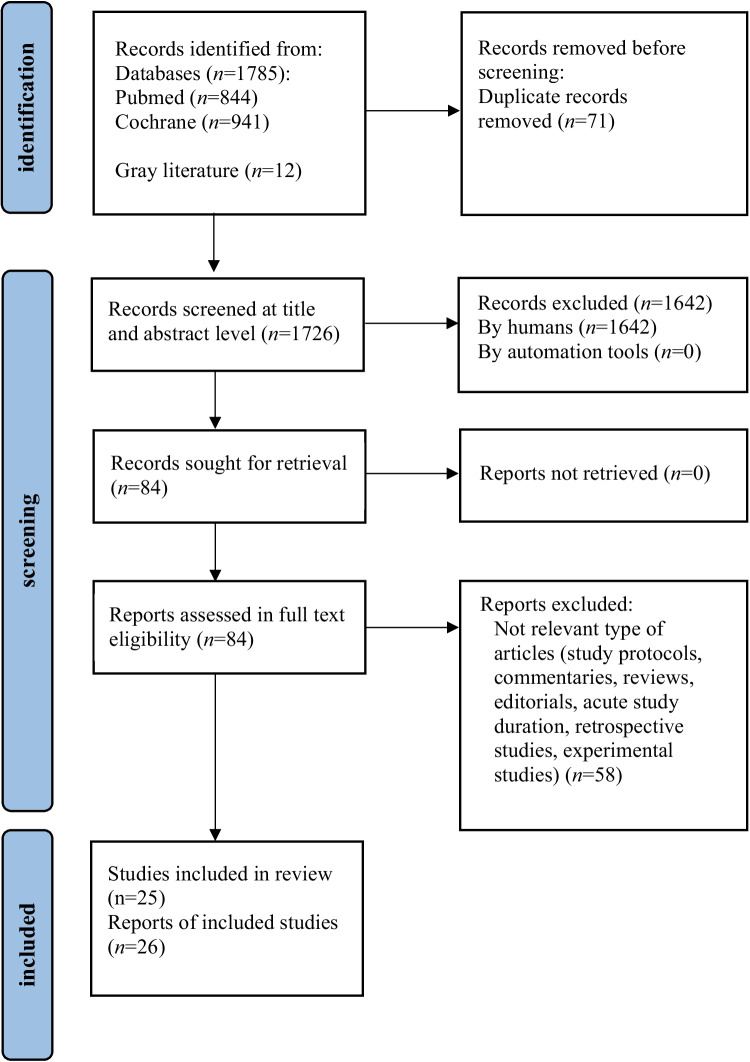
Preferred Reporting Items for Systematic reviews and Meta-Analyses (PRISMA) flow chart for the identification, inclusion, and exclusion of studies

The flow diagram of the study selection process is shown in Fig. 1. Twenty-six reports from 25 trials comprising a total of 3224 patients with T1D reported at least one of the predefined outcomes of interest and were included in the systematic review. Study and participants’ baseline characteristics are presented in Supplementary Table 1.

Although we did not use age restrictions, only studies in adults were available. The most commonly studied GLP-1RA was liraglutide (*N* = 16 trials) [18–34], followed by exenatide (*N* = 6) [35–40], lixisenatide (*N* = 1) [41], semaglutide (*N* = 1) [42], and dulaglutide (*N* = 1) [43]. Mean T1D duration was 17 years, including three studies that recruited patients with a very recent diagnosis of T1D (from 33 days to 10 weeks from diagnosis) [26, 27, 38]. Mean age of the included participants was 40.5 years, their mean HbA_1c_ being 7.9% and BMI 26.6 kg/m^2^. Study duration ranged from 4 weeks to 1 year. Three studies were available only as conference abstracts [26, 33, 34].

### Glycemic control

**Fig. 2:**
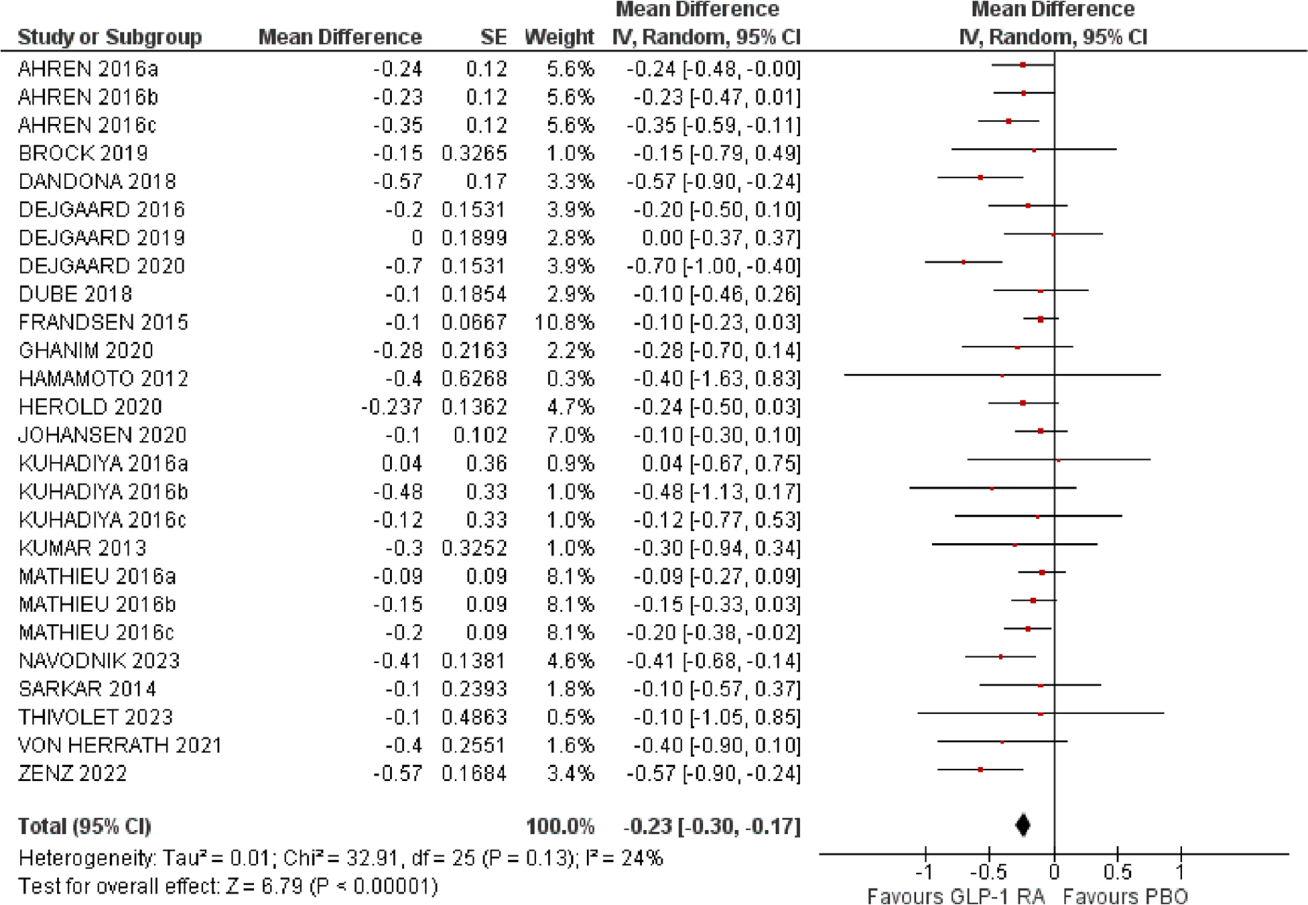
Effect of GLP-1RA compared with placebo on HbA_1c_ (%). * a: liraglutide 0.6 mg, b: liraglutide 1.2 mg, c: liraglutide 1.8 mg. PBO: placebo, CI: confidence interval

Twenty studies with 3095 participants had a duration of at least 12 weeks and were included in the analysis for the change in HbA_1c_ [18, 20–28, 31–35, 37, 38, 40, 42, 43]. GLP-1RA improved glycemic control by reducing HbA_1c_ compared to placebo (MD −0.23%, 95% CI −0.30 to −0.17, Ι^2^ 24%) (Fig. 2). The risk of bias was deemed low only for six trials, there were some concerns for five trials, and in nine trials the risk of bias was considered high (Supplementary Material). We then performed a pre-specified subgroup analysis based on baseline C-peptide levels. Nine studies included either patients with new onset of T1D or with detectable C-peptide levels [21, 22, 26, 31, 34, 35, 43] and were considered having preserved β-cell function. In patients with preserved β-cell function, the addition of a GLP-1RA improved HbA_1c_ compared to placebo to a larger extent than in patients without residual β-cell function (MD −0.42%, 95% CI −0.57 to −0.26, Ι^2^ 60% *vs* MD −0.17%, 95% CI −0.22 to −0.12, test for subgroup differences *p* = 0.003) (Supplementary Fig. 1). We did not identify asymmetry in the Funnel plot (Supplementary Material, Supplementary Fig. 2).

**Fig. 3:**
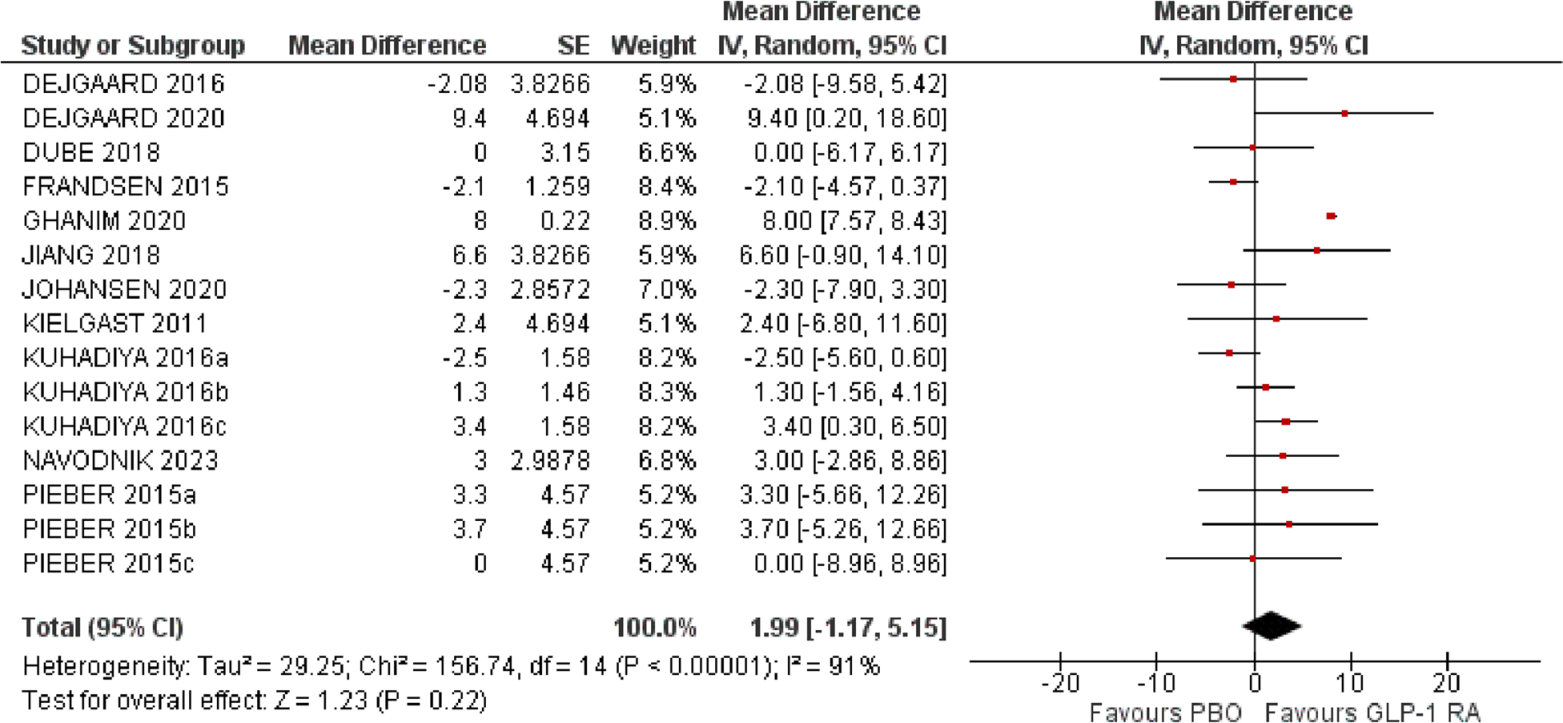
Effect of GLP-1RA compared with placebo on time-in-range (TIR) (%). * a: liraglutide 0.6 mg, b: liraglutide 1.2 mg, c: liraglutide 1.8 mg. PBO: placebo, CI: confidence interval

Eleven studies involving 549 patients reported data on TIR. Most studies defined TIR as target interstitial glucose levels between 3.9–10.0 mmol/L, except for three studies [25, 28, 32] which used a more stringent interstitial glucose range between (3.8–7.8 mmol/L, 3.9–8.9 mmol/L and 3.8–8.8 mmol/L, respectively) to define TIR. Most studies presented TIR data as %. Few studies presented TIR data as hours per day and in these studies TIR was calculated as % after multiplying by 100 and dividing by 24. Duration of CGM recordings also varied among studies (Supplementary Material). Use of a GLP-1RA did not significantly affect TIR (MD 1.99%, 95% CI −1.17 to 5.15, Ι^2^ 91%) (Fig. 3).

### Body weight

**Fig. 4:**
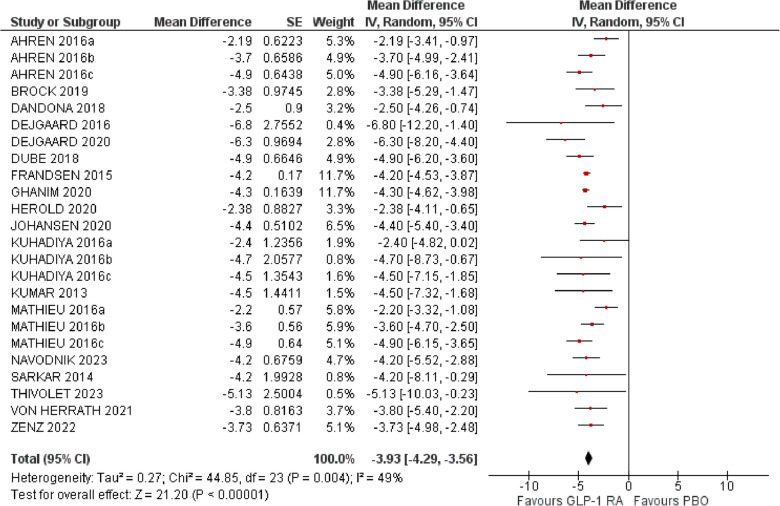
Effect of GLP-1RA compared with placebo on body weight (kg). *a: liraglutide 0.6 mg, b: liraglutide 1.2 mg, c: liraglutide 1.8 mg. PBO: placebo, CI: confidence interval

Twenty studies involving 3095 patients were included in the analysis for the change in body weight [18, 20–28, 31–35, 37, 38, 40, 42, 43]. Use of GLP-1 RAs reduced body weight (MD −3.93 kg, 95% CI −4.29 to −3.56, Ι^2^ 49%) (Fig. 4).

### Total insulin dose

Fourteen studies reported total insulin dose in U/day [18, 20–23, 26, 28, 30–32, 34, 36, 41, 42], five studies reported it in U/kg [25, 27, 29, 37, 43], and four studies reported both values [24, 35, 38, 40]. Wherever possible, calculation of both TID (U/day) and TID per kg was made. Data from 22 studies that involved 3151 patients were available for TID expressed in U/day [18, 20–23, 25–28, 30–32, 34–38, 40–44]. GLP-1RA decreased total insulin dose compared to placebo (MD −5.74 U/day, 95% CI −7.30 to −4.19, Ι^2^ 71%) (Supplementary Fig. 3A). Similar results were obtained when calculating total insulin dose per kg; GLP-1RA decreased total insulin dose per kg compared to placebo (MD −0.06 U/kg, 95% CI −0.09 to −0.03, Ι^2^ 79%) (Supplementary Fig. 3B).

### Severe hypoglycemia

**Fig. 5:**
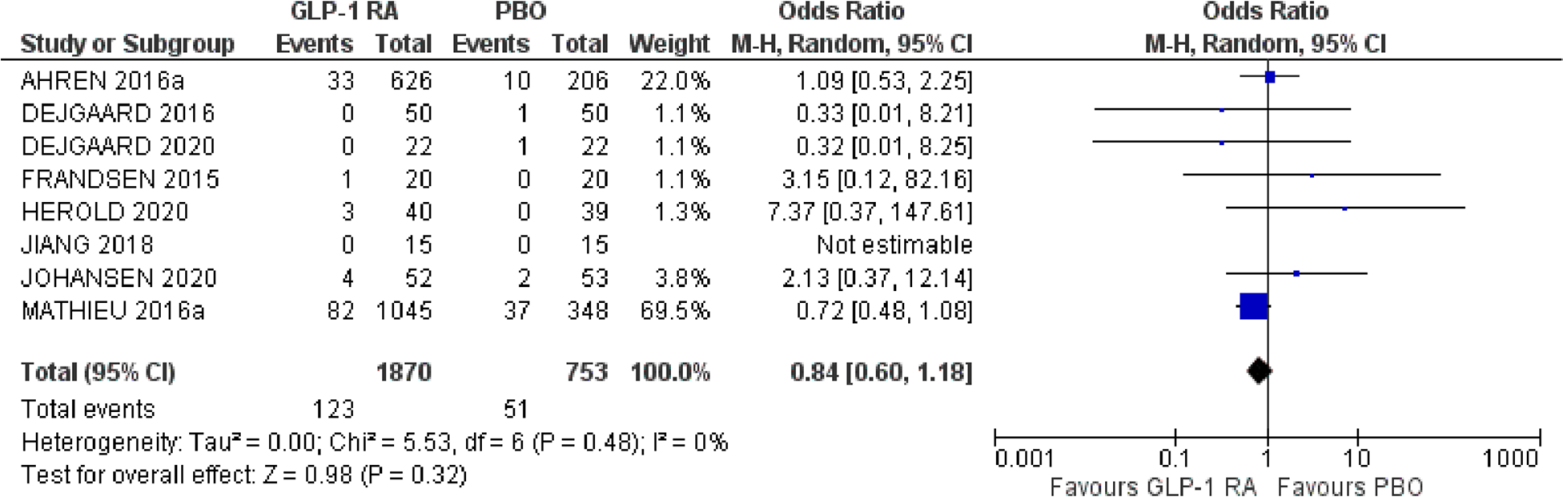
Effect of GLP-1RA compared with placebo on the incidence of severe hypoglycemia. * a: liraglutide 0.6 mg. PBO: placebo, CI: confidence interval

Thirteen studies with 2882 patient reported the number of patients with at least one episode of severe hypoglycemia [18, 21–24, 26–28, 30, 34–36, 40]. Of these, six studies [26–28, 30, 34, 36] had 0 events in both arms and thus the odds ratio for these studies could not be calculated. Concerning the remaining eight studies involving 2623 patients, it was shown that GLP-1RA did not affect the risk of having an episode of severe hypoglycemia compared to placebo (OR 0.84, 95% CI 0.60 to 1.18, Ι^2^ 0%) (Fig. 5).

### Individual study results for the effects of GLP-1RA on C-peptide and glucagon levels

#### Changes in C-peptide following treatment with GLP-1RA

##### Studies reporting an increase in C-peptide

Kumar et al. examined the effects of exenatide in 12 patients with T1D, randomized to either receive no additional treatment or exenatide for 1 year [38]. While stimulated C-peptide levels numerically declined in the insulin-only group, they exhibited a slight increase in the exenatide-treated cohort. Similarly, Thivolet et al. conducted a standard mixed-meal tolerance test (MMTT) at baseline and after 24 weeks of dulaglutide treatment, observing a numerical, albeit non-significant, increase in C-peptide area under the curve (AUC) [43].

##### Studies reporting a decrease in C-peptide

Hamamoto et al. utilized glucagon- and arginine-stimulation tests at baseline and after 52 weeks of liraglutide therapy, assessing β-cell function. By the study’s conclusion, C-peptide responses to both stimuli showed a slight reduction in both the liraglutide and insulin-only groups [34]. Von Herrath et al. investigated MMTT-stimulated C-peptide levels following 54 weeks of liraglutide treatment, reporting comparable reductions in both the liraglutide and placebo groups [27].

##### Studies reporting no significant change in C-peptide

Herold et al. conducted a MMTT at baseline and at weeks 12 and 24, specifically evaluating C-peptide-positive patients. Their findings indicated no differences in C-peptide levels either at baseline or following treatment with exenatide [35]. Finally, in the abstract by Dejgaard et al., liraglutide sustained C-peptide secretion at end of treatment (52 weeks) in patients with newly diagnosed T1D. However, 6 weeks post-treatment, stimulated C-peptide levels did not differ between the liraglutide and placebo groups [26].

Taken together, these findings suggest that GLP-1RAs have negligible and non-significant effects on the preservation of C-peptide levels in T1D.

#### Effects of GLP-1RA treatment on glucagon levels

##### Studies reporting a decrease in glucagon levels

Kielgast et al. investigated the effects of liraglutide in 19 patients with T1D. Participants underwent a MMTT and a subsequent 45-min exercise test. While fasting glucagon levels remained unchanged, postprandial glucagon levels during the MMTT were significantly reduced. However, during exercise, plasma glucagon levels increased similarly in both groups [29]. Kuhadiya et al. also performed a MMTT and observed a dose-dependent reduction in postprandial glucagon levels following treatment with liraglutide [28].

Ballav et al. assessed the effects of lixisenatide over 4 weeks. During the MMTT, both glucose and glucagon AUC were lower in the lixisenatide group compared to placebo. However, plasma glucagon levels during the hypoglycemic clamp remained similar between groups [41].

##### Studies reporting no change in glucagon levels

Dejgaard et al. observed no significant change in glucagon AUC during the MMTT in patients receiving liraglutide for 24 weeks compared to those receiving placebo [24]. Similarly, Herold et al. reported no significant differences in glucagon AUC or peak glucagon values during a MMTT at baseline and weeks 12 and 24 of treatment with exenatide [35].

Frandsen et al. conducted a 30-min euglycemic clamp followed by hypoglycemia induction. When plasma glucose values reached 2.5–2.8 mmol/L, insulin infusion was discontinued and a liquid meal was administered to restore glucose levels. The authors reported no differences in mean plasma glucagon values, incremental glucagon AUC, or peak glucagon levels between the groups receiving liraglutide or placebo [19].

Thivolet et al. performed a MMTT at baseline and after 24 weeks of treatment with dulaglutide and reported no significant changes in median glucagon AUC [43]. Likewise, Van Meijel et al. performed hyperinsulinemic euglycemic clamps followed by hypoglycemic clamps (nadir 2.5 mmol/L). At the end of each treatment period they found no differences in plasma glucagon values at baseline, euglycemia, or hypoglycemia between the exenatide and placebo groups [39].

Taken together, while the effects of GLP-1RA therapy on glucagon regulation during a MMTT appear heterogeneous, all studies consistently show that treatment with GLP-1RA does not reduce glucagon levels during hypoglycemia when its counter-regulatory action is essential.

## Discussion

The primary aim of the present study was to assess whether addition of a GLP-1RA on top of insulin treatment results in improvement of HbA_1c_ in patients with T1D. Additionally, we assessed the effects of the addition of a GLP-1RA on body weight, total insulin dose, TIR, and incidence of severe hypoglycemia.

We found that treatment with liraglutide, exenatide, lixisenatide, dulaglutide, or semaglutide resulted in a small improvement in glycemic control, with no difference between individual GLP-1 RAs. The decrease in HbA_1c_ is smaller than that which has been previously reported in patients with T2D receiving GLP-1RA, which ranges from −0.9 to −1.4% [45, 46]. On the one hand, this smaller improvement in HbA_1c_ can be explained by the fact that patients with T1D have a smaller reserve of β-cells and, thus, the main action of GLP-1RAs as insulin secretagogues is curtailed. This conclusion is also supported by the subgroup analysis which showed a significantly larger effect in decreasing HbA_1c_ in patients with preserved C-peptide reserve compared to those without preserved C-peptide reserve. Additionally, the smaller decrease in HbA_1c_ in patients with T1D compared to patients with T2D after the addition of a GLP-1RA has been attributed to a concomitant decrease in prandial insulin dose to avoid post-prandial hypoglycemia [47]. Although in the present meta-analysis we did not specifically address prandial insulin doses, total insulin dose was decreased in patients receiving a GLP-1RA. Moreover, most studies included in this meta-analysis reported that the decrease in total insulin dose was due to a greater reduction in bolus insulin doses [18, 21–25, 36, 37, 40, 41], while basal insulin dose remained unchanged in most trials and decreased significantly only in the study by Kuhadiya and colleagues [28].

We also observed a modest decrease in body weight in patients receiving a GLP-1RA compared to placebo. This finding could be attributed both to the mechanisms of action of GLP-1RA (delayed gastric emptying, increased satiety, and eventual effects in the central control of appetite [48]) and to the decreased need for insulin, as insulin is a major anabolic hormone. The current obesity epidemic is also affecting patients with T1D who were typically considered as mostly lean and insulin sensitive. Indeed, the term “double diabetes” has been coined to describe the presence of insulin resistance in patients with T1D [49]. On the one hand, obesity and insulin resistance would exert additional stress on the remaining β-cells, which are already facing insults due to autoimmunity thereby accelerating their demise [50]. On the other hand, insulin resistance would result in even larger needs for insulin and, thus, a vicious cycle of weight gain and increased total insulin dose would be established. Importantly, a large study in 31,119 patients with T1D has shown that “double diabetes” is an independent risk factor for development of both microvascular and macrovascular complications [51]. Hence, use of GLP-1RA in patients with T1D may prevent the occurrence or mitigate “double diabetes” in patients with T1D thereby improving their clinical outcomes.

In the few studies that also reported data on TIR, addition of a GLP-1RA seemed also to have a beneficial effect on TIR, but this did not reach statistical significance. This lack of effect might be attributed to several reasons. Firstly, only a small number of studies evaluated this outcome. Moreover, we noticed heterogeneity in the CGM recordings of the different studies regarding the definition of TIR and the duration of CGM, the latter ranging from 3 days to 12 weeks of recording. Another important aspect was that most studies used blinded CGM, whereas two studies did not mention CGM blinding [32, 36]. This is an important factor if we consider that CGM per se may improve glycemic control; thus, unblinded CGM studies bear an inherent bias [52]. Of note, some studies reported profiles of frequent self-monitoring of blood glucose and recently Beck and colleagues used seven-point blood glucose profiles from the DCCT trial to calculate an estimated TIR [53]. As we had not specified in our meta-analysis protocol that seven-point blood glucose profiles would be used to estimate TIR, these studies were not included in the data synthesis. Overall, our systematic review highlights a knowledge gap regarding the effects of GLP-1RA addition in patients with T1D on TIR and may help guide future research. Additional original studies with larger number of patients will be needed to clarify whether addition of GLP-1RA may effectively improve TIR. This is clinically important as TIR has been shown to be a stronger predictor of microvascular complications than HbA_1c_ [54].

A concern regarding the use of a GLP-1RA in patients with T1D in clinical practice would be the risk of hypoglycemia, as GLP-1RAs not only enhance endogenous insulin secretion but also suppress glucagon release [55], a major counter-regulatory hormone. We found that severe hypoglycemia occurred at a similar rate in both patients receiving a GLP-1RA or placebo. Due to marked differences in the outcomes of C-peptide and glucagon reported in the various trials, we were not able to synthesize these results and therefore presented the data in a qualitative manner. Nonetheless, based on the limited number of studies assessing this outcome, it emerges that the addition of GLP-1RA does not prevent progressive C-peptide loss. As far as plasma glucagon levels are concerned, some studies showed that during a MMTT glucagon AUC is decreased in patients treated with a GLP-1RA [28, 29, 41]. Importantly, however, when hypoglycemia was induced there were no differences in glucagon levels in patients receiving GLP-1RA compared to placebo [19, 39, 41]. Thus, patients receiving a GLP-1RA do not have inadequate glucagon secretion in response to hypoglycemia and are consequently not exposed to an increased risk for severe hypoglycemia. This conclusion is in line with the current result of similar incidence of severe hypoglycemia in patients receiving GLP-1RA or placebo.

A long-debated question in diabetes care is the threshold of HbA_1c_ change that may be considered clinically significant. Here, we observed a 0.23% reduction in HbA_1c_ which may be considered fairly small and is smaller than what has previously been described for GLP-1RA in patients with T2D. Arguably, however, any HbA_1c_ reduction in patients with diabetes not accompanied by an increase in hypoglycemia may be considered beneficial for patients. The 3.9 kg reduction in body weight is close to the recommended target of at least 5% reduction following the addition of a weight loss agent in people with obesity [56].

### Implications for clinical practice and future research

From a clinical standpoint, the findings of this systematic review and meta-analysis support the use of GLP-1RA in patients with T1D, particularly those with overweight or obesity. The addition of GLP-1RA in patients with T1D not only improves glycemic control and promotes weight loss but could also provide cardiovascular and renal protection. Future RCTs assessing renal and cardiovascular outcomes in patients with T1D would be valuable to further establish their benefits. Additionally, large-scale cardiovascular outcome trials will be necessary before GLP-1 RA can be incorporated into treatment guidelines for T1D.

### Strengths and limitations

Our findings regarding the favorable effect of GLP-1RA on glycemic control, body weight, and total insulin dose are in line with the findings of another recent meta-analysis which reported results for liraglutide, exenatide, lixisenatide, and albiglutide and showed that addition of a GLP-1RA on top of insulin treatment decreased HbA_1c_ by 0.21%, decreased body weight by 3.78 kg and decreased TID by 5.84 U/day while not increasing the risk of severe hypoglycemia [12]. The latter meta-analysis included 24 studies, including one study on albiglutide [57] which we chose not to include by study protocol as this drug has been withdrawn from the market [58]. Moreover, our systematic review included two recently published RCTs using the once-weekly GLP-1RAs dulaglutide and semaglutide [42, 43]. Finally, the novelty of our meta-analysis lies in assessing for the first time, to our knowledge, the impact of the addition of GLP-1RA on TIR as well as on C-peptide and glucagon levels, outcomes of both important clinical and pathophysiological relevance.

Our study has some limitations. First, we detected significant heterogeneity for some of the outcomes of interest, namely, for total insulin dose and TIR, whereas heterogeneity was small for the primary outcome. This is probably to be attributed to the fact that HbA_1c_ was the primary outcome in most of the trials included and, therefore, the change in HbA_1c_ was described in the original publications in detail. The clinical outcome of body weight was also sufficiently reported in most trials. On the contrary, measures of dispersion were often missing for the other outcomes and had to be retrieved either from figures or to be imputed. This is unlikely to have had a significant impact on the meta-analysis results as mean differences were provided for all studies. Indeed, a sensitivity analysis for the primary outcome, excluding studies where imputation methods were required, yielded similar results (Supplementary Material). Moreover, patients in the included trials received either multiple daily insulin injections or continuous subcutaneous insulin infusion and had varying β-cell function, which may have contributed to the heterogeneity in total insulin dose outcomes. Additionally, most of the included studies had small sample sizes and were of relatively small duration. Finally, many of the included trials were deemed to be of high concern for risk of bias assessment, making the quality of evidence relatively poor.

To conclude, addition of a GLP-1RA on top of insulin treatment results in improvements in glycemic control (HbA_1c_) and body weight while reducing total daily insulin needs. At the same time, patients receiving GLP-1RA are not exposed to an increased risk for severe hypoglycemia. These results show promise for the use of the GLP-1RA approach in patients with T1D. In contrast, addition of a GLP-1RA on top of insulin treatment does not appear to preserve C-peptide levels and further research is warranted to evaluate its impact on TIR.

## Supplementary Information

Below is the link to the electronic supplementary material.

Supplementary file1 (DOCX 292 KB)

Supplementary file2 (DOCX 21 KB)

## Data Availability

The data that support the findings of this study are available from the corresponding author upon reasonable request.

## References

[1] DiMeglio LA, Evans-Molina C, Oram RA (2018) Type 1 diabetes. Lancet 391:2449–2462. 10.1016/S0140-6736(18)31320-5 29916386 10.1016/S0140-6736(18)31320-5 PMC6661119

[2] Butler AE, Galasso R, Meier JJ, Basu R, Rizza RA, Butler PC (2007) Modestly increased beta cell apoptosis but no increased beta cell replication in recent-onset type 1 diabetic patients who died of diabetic ketoacidosis. Diabetologia 50:2323–2331. 10.1007/s00125-007-0794-x 17805509 10.1007/s00125-007-0794-x

[3] Madsbad S (1983) Prevalence of residual B cell function and its metabolic consequences in type 1 (insulin-dependent) diabetes. Diabetologia 24:141–147. 10.1007/BF00250151 6341142 10.1007/BF00250151

[4] Löhr M, Klöppel G (1987) Residual insulin positivity and pancreatic atrophy in relation to duration of chronic type 1 (insulin-dependent) diabetes mellitus and microangiopathy. Diabetologia 30:757–762. 10.1007/BF00275740 3322901 10.1007/BF00275740

[5] Madsbad S, Krarup T, Reguer L, Faber OK, Binder C (1981) Effect of strict blood glucose control on residual B-cell function in insulin-dependent diabetics. Diabetologia 20:530–534. 10.1007/BF00252760 7026327 10.1007/BF00252760

[6] Effect of Intensive Therapy on Residual Beta-Cell Function in Patients with Type 1 Diabetes in the Diabetes Control and Complications Trial. A Randomized, Controlled Trial. The Diabetes Control and Complications Trial Research Group. (1998) Ann Intern Med 128: 517–523. 10.7326/0003-4819-128-7-199804010-00001. 10.7326/0003-4819-128-7-199804010-00001 9518395

[7] Brown RJ, Sinaii N, Rother KI (2008) Too much glucagon, too little insulin: time course of pancreatic islet dysfunction in new-onset type 1 diabetes. Diabetes Care 31:1403–1404. 10.2337/dc08-0575 18594062 10.2337/dc08-0575 PMC2453684

[8] Taylor PN, Collins KS, Lam A et al (2023) C-Peptide and Metabolic Outcomes in Trials of Disease Modifying Therapy in New-Onset Type 1 Diabetes: An Individual Participant Meta-Analysis. Lancet Diabetes Endocrinol 11:915–925. 10.1016/S2213-8587(23)00267-X 37931637 10.1016/S2213-8587(23)00267-X

[9] De Marinis YZ, Salehi A, Ward CE et al (2010) GLP-1 inhibits and adrenaline stimulates glucagon release by differential modulation of N- and L-type Ca2+ channel-dependent exocytosis. Cell Metab 11:543–553. 10.1016/j.cmet.2010.04.007 20519125 10.1016/j.cmet.2010.04.007 PMC4310935

[10] Andreasen CR, Andersen A, Knop FK, Vilsbøll T (2021) Understanding the place for GLP-1RA therapy: translating guidelines for treatment of type 2 diabetes into everyday clinical practice and patient selection. Diabetes Obes Metab 23(Suppl 3):40–52. 10.1111/dom.14500 34519400 10.1111/dom.14500

[11] Tan X, Pan X, Wu X, Zheng S, Chen Y, Liu D, Zhang X (2023) Glucagon-like peptide-1 receptor agonists as add-on therapy to insulin for type 1 diabetes mellitus. Front Pharmacol 14:975880 38249345 10.3389/fphar.2023.975880 PMC10797415

[12] Park J, Ntelis S, Yunasan E, Downton KD, Yip TC-F, Munir KM, Haq N (2023) Glucagon-like peptide 1 analogues as adjunctive therapy for patients with type 1 diabetes: an updated systematic review and meta-analysis. J Clin Endocrinol Metab 109:279–292. 10.1210/clinem/dgad471 37561012 10.1210/clinem/dgad471

[13] Page MJ, McKenzie JE, Bossuyt PM et al (2021) The PRISMA 2020 statement: an updated guideline for reporting systematic reviews. J Clin Epidemiol 134:178–189. 10.1016/j.jclinepi.2021.03.001 33789819 10.1016/j.jclinepi.2021.03.001

[14] Higgins J, Thomas J, Chandler J, Cumpston M, Li T, Page M, VA W (editors) (Updated August 2023) Cochrane Handbook for Systematic Reviews of Interventions Version 6.4 Available online: www.training.cochrane.org/handbook.

[15] Cates C (2015) Multiple-Arm Trial Data: Using a Corrected Standard Error for GIV Analyses. In *Cochrane Colloquium*; pp. 1–327.

[16] Sterne JAC, Savović J, Page MJ et al (2019) RoB 2: a revised tool for assessing risk of bias in randomised trials. BMJ 366:l4898. 10.1136/bmj.l4898 31462531 10.1136/bmj.l4898

[17] Deeks J, Higgins J, Altman D (2023) Chapter 10: Analysing Data and Undertaking Meta-Analyses. In: Higgins JPT, Thomas J, Chandler J, et Al., Eds. Cochrane Handbook for Systematic Reviews of Interventions. Version 6.4. The Cochrane Collaboration. Accessed April 21, 2024.

[18] Frandsen CS, Dejgaard TF, Holst JJ, Andersen HU, Thorsteinsson B, Madsbad S (2015) Twelve-week treatment with liraglutide as add-on to insulin in normal-weight patients with poorly controlled type 1 diabetes: a randomized, placebo-controlled, double-blind parallel study. Diabetes Care 38:2250–2257. 10.2337/dc15-1037 26486191 10.2337/dc15-1037

[19] Frandsen CS, Dejgaard TF, Andersen HU, Holst JJ, Hartmann B, Thorsteinsson B, Madsbad S (2017) Liraglutide as adjunct to insulin treatment in type 1 diabetes does not interfere with glycaemic recovery or gastric emptying rate during hypoglycaemia: a randomized, placebo-controlled, double-blind, parallel-group study. Diabetes Obes Metab 19:773–782. 10.1111/dom.12830 27868372 10.1111/dom.12830

[20] Brock C, Hansen CS, Karmisholt J, Møller HJ, Juhl A, Farmer AD, Drewes AM, Riahi S, Lervang HH, Jakobsen PE et al (2019) Liraglutide treatment reduced interleukin‐6 in adults with type 1 diabetes but did not improve established autonomic or polyneuropathy. Br J Clin Pharmacol 85:2512–2523. 10.1111/bcp.14063 31338868 10.1111/bcp.14063 PMC6848951

[21] Mathieu C, Zinman B, Hemmingsson JU, Woo V, Colman P, Christiansen E, Linder M, Bode B (2016) Efficacy and safety of liraglutide added to insulin treatment in type 1 diabetes: the ADJUNCT ONE treat-to-target randomized trial. Diabetes Care 39:1702–1710. 10.2337/dc16-0691 27506222 10.2337/dc16-0691

[22] Ahrén B, Hirsch IB, Pieber TR et al (2016) Efficacy and safety of liraglutide added to capped insulin treatment in subjects with type 1 diabetes: the ADJUNCT TWO randomized trial. Diabetes Care 39:1693–1701. 10.2337/dc16-0690 27493132 10.2337/dc16-0690

[23] Dejgaard TF, Schmidt S, Frandsen CS, Vistisen D, Madsbad S, Andersen HU, Nørgaard K (2020) Liraglutide reduces hyperglycaemia and body weight in overweight, dysregulated insulin-pump-treated patients with type 1 diabetes: the Lira pump trial-a randomized, double-blinded, placebo-controlled trial. Diabetes Obes Metab 22:492–500. 10.1111/dom.13911 31696598 10.1111/dom.13911

[24] Dejgaard TF, Frandsen CS, Hansen TS et al (2016) Efficacy and Safety of Liraglutide for Overweight Adult Patients with Type 1 Diabetes and Insufficient Glycaemic Control (Lira-1): A Randomised, Double-Blind. Placebo-Controlled Trial Lancet Diabetes Endocrinol 4:221–232. 10.1016/S2213-8587(15)00436-2 26656289 10.1016/S2213-8587(15)00436-2

[25] Ghanim H, Batra M, Green K, Abuaysheh S, Hejna J, Makdissi A, Borowski R, Kuhadiya ND, Chaudhuri A, Dandona P (2020) Liraglutide treatment in overweight and obese patients with type 1 diabetes: a 26-week randomized controlled trial; mechanisms of weight loss. Diabetes Obes Metab 22:1742–1752. 10.1111/dom.14090 32424935 10.1111/dom.14090

[26] Dejgaard TF, Frandsen CS, Kielgast U, et al. (2019) 59-OR: Liraglutide 1, Preserved Insulin Secretion in Adults with Newly Diagnosed Type 1):59-OR., Diabetes: The NewLira Trial. *Diabetes 68*: 59-OR.

[27] von Herrath M, Bain SC, Bode B et al (2021) Anti-Interleukin-21 Antibody and Liraglutide for the Preservation of β-Cell Function in Adults with Recent-Onset Type 1 Diabetes: A Randomised, Double-Blind, Placebo-Controlled, Phase 2 Trial. Lancet Diabetes Endocrinol 9:212–224. 10.1016/S2213-8587(21)00019-X 33662334 10.1016/S2213-8587(21)00019-X

[28] Kuhadiya ND, Dhindsa S, Ghanim H et al (2016) Addition of liraglutide to insulin in patients with type 1 diabetes: a randomized placebo-controlled clinical trial of 12 weeks. Diabetes Care 39:1027–1035. 10.2337/dc15-1136 27208343 10.2337/dc15-1136 PMC5864130

[29] Kielgast U, Krarup T, Holst JJ, Madsbad S (2011) Four weeks of treatment with Liraglutide reduces insulin dose without loss of glycemic control in type 1 diabetic patients with and without residual beta-cell function. Diabetes Care 34:1463–1468. 10.2337/dc11-0096 21593296 10.2337/dc11-0096 PMC3120168

[30] Pieber TR, Deller S, Korsatko S, Jensen L, Christiansen E, Madsen J, Heller SR (2015) Counter-regulatory hormone responses to hypoglycaemia in people with type 1 diabetes after 4 weeks of treatment with liraglutide adjunct to insulin: a randomized, placebo-controlled, double-blind, crossover trial. Diabetes Obes Metab 17:742–750. 10.1111/dom.12473 25855340 10.1111/dom.12473

[31] Zenz S, Regittnig W, Boulgaropoulos B et al (2022) Effect of liraglutide treatment on whole-body glucose fluxes in C-peptide-positive type 1 diabetes during hypoglycemia. J Clin Endocrinol Metab 107:e3583–e3593. 10.1210/clinem/dgac369 35833597 10.1210/clinem/dgac369

[32] Dubé MC, D’Amours M, Weisnagel SJ (2018) Beyond Glycaemic Control: A Cross-over, Double-Blinded, 24-Week Intervention with Liraglutide in Type 1 Diabetes. Diabetes Obes Metab 20:178–184. 10.1111/dom.13063 28722271 10.1111/dom.13063

[33] Dandona P, Ghanim H, Kuhadiya ND, Shah T, Hejna JM, Makdissi A, Batra M, Chaudhuri A (2018) Liraglutide as an additional treatment to insulin in patients with type 1 diabetes mellitus-a 52-week randomized double-blinded placebo-controlled clinical trial. Diabetes. 10.2337/db18-3-LB

[34] Abstracts of the 48th EASD (European Association for the Study of Diabetes) Annual Meeting of the European Association for the Study of Diabetes. 2012(October), pp. 1–5 (2012) Berlin, Germany. Diabetologia 55:S300 10.1007/s00125-012-2688-9 22918257

[35] Herold KC, Reynolds J, Dziura J et al (2020) Exenatide extended release in patients with type 1 diabetes with and without residual insulin production. Diabetes Obes Metab 22:2045–2054. 10.1111/dom.14121 32573927 10.1111/dom.14121 PMC8009602

[36] Jiang LL, Wang SQ, Ding B et al (2018) The effects of add-on exenatide to insulin on glycemic variability and hypoglycemia in patients with type 1 diabetes mellitus. J Endocrinol Invest 41:539–547. 10.1007/s40618-017-0765-0 29032494 10.1007/s40618-017-0765-0

[37] Sarkar G, Alattar M, Brown RJ, Quon MJ, Harlan DM, Rother KI (2014) Exenatide treatment for 6 months improves insulin sensitivity in adults with type 1 diabetes. Diabetes Care 37:666–670. 10.2337/dc13-1473 24194508 10.2337/dc13-1473 PMC3931382

[38] Hari Kumar KV, Shaikh A, Prusty P (2013) Addition of exenatide or sitagliptin to insulin in new onset type 1 diabetes: a randomized, open label study. Diabetes Res Clin Pract 100:e55–e58. 10.1016/j.diabres.2013.01.020 23490599 10.1016/j.diabres.2013.01.020

[39] Van Meijel L, Rooijackers HM, Tack CJ, De Galan BE (2019) Effect of the GLP-1 Receptor Agonist Exenatide on Awareness of Hypoglycaemia in Patients with Type 1 Diabetes and Impaired Awareness of Hypoglycaemia. Diabetes Technol Ther 21:A132–A133. 10.1089/dia.2019.2525.abstracts 10.1210/jc.2019-00087 30958544

[40] Johansen NJ, Dejgaard TF, Lund A et al (2020) Efficacy and Safety of Meal-Time Administration of Short-Acting Exenatide for Glycaemic Control in Type 1 Diabetes (MAG1C): A Randomised, Double-Blind. Placebo-Controlled Trial Lancet Diabetes Endocrinol 8:313–324. 10.1016/S2213-8587(20)30030-9 32135138 10.1016/S2213-8587(20)30030-9

[41] Ballav C, Dhere A, Kennedy I et al (2020) Lixisenatide in type 1 diabetes: a randomised control trial of the effect of lixisenatide on post-meal glucose excursions and glucagon in type 1 diabetes patients. Endocrinology, Diabetes & Metabolism 3:e00130. 10.1002/edm2.130 10.1002/edm2.130 PMC7375047 32704555

[42] Navodnik MP, Janež A, Žuran I (2023) The effect of additional treatment with Empagliflozin or Semaglutide on endothelial function and arterial stiffness in subjects with type 1 diabetes mellitus-ENDIS study. Pharmaceutics 15:1945. 10.3390/pharmaceutics15071945 37514131 10.3390/pharmaceutics15071945 PMC10385568

[43] Thivolet C, Larger E, Cariou B (2023) Dulaglutide and insulin microsecretion in people with type 1 diabetes (DIAMOND-GLP-1): a randomized double-blind placebo-controlled trial. Diabetes Metab 49:101433. 10.1016/j.diabet.2023.101433 36781064 10.1016/j.diabet.2023.101433

[44] Frandsen CS, Dejgaard TF, Madsbad S (2016) Non-Insulin Drugs to Treat Hyperglycaemia in Type 1 Diabetes Mellitus. Lancet Diabetes Endocrinol 4:766–780. 10.1016/S2213-8587(16)00039-5 26969516 10.1016/S2213-8587(16)00039-5

[45] Vanderheiden A, Harrison LB Warshauer JT, et al. (2016) Mechanisms of Action of Liraglutide in Patients With Type 2 Diabetes Treated With High-Dose Insulin. J Clin Endocrinol Metab 101: 1798‐1806. 10.1210/jc.2015-3906. 10.1210/jc.2015-3906 26909799

[46] Hu S, Su X, Fan G (2023) Efficacy and tolerability of the subcutaneous semaglutide for type 2 diabetes patients: an updated systematic review and meta-analysis. Diabetol Metab Syndr 15:218. 10.1186/s13098-023-01195-7 37891683 10.1186/s13098-023-01195-7 PMC10612199

[47] Nauck MA, Meier JJ (2020) GLP-1 Receptor Agonists in Type 1 Diabetes: A MAG1C Bullet? *Lancet*. Diabetes Endocrinol 8:262–264. 10.1016/S2213-8587(20)30043-7 10.1016/S2213-8587(20)30043-7 32135134

[48] Halawi H, Khemani D, Eckert D et al (2017) Effects of Liraglutide on Weight, Satiation, and Gastric Functions in Obesity: A Randomised Placebo-Controlled Pilot Trial. Lancet Gastroenterol Hepatol 2:890–899. 10.1016/S2468-1253(17)30285-6 28958851 10.1016/S2468-1253(17)30285-6

[49] Teupe B, Bergis K (1991) Epidemiological Evidence for “Double Diabetes.” Lancet 337:361–362 1671252 10.1016/0140-6736(91)90988-2

[50] Buzzetti R, Zampetti S, Pozzilli P (2020) Impact of obesity on the increasing incidence of type 1 diabetes. Diabetes Obes Metab 22:1009–1013. 10.1111/dom.14022 32157790 10.1111/dom.14022

[51] Merger SR, Kerner W, Stadler M, Zeyfang A, Jehle P, Müller-Korbsch M, Holl RW (2016) Prevalence and comorbidities of double diabetes. Diabetes Res Clin Pract 119:48–56. 10.1016/j.diabres.2016.06.003 27449710 10.1016/j.diabres.2016.06.003

[52] Bode B, Beck RW, Xing D et al (2009) Sustained benefit of continuous glucose monitoring on A1C, glucose profiles, and hypoglycemia in adults with type 1 diabetes. Diabetes Care 32:2047–2049. 10.2337/dc09-0846 19675193 10.2337/dc09-0846 PMC2768224

[53] Beck RW, Bergenstal RM, Riddlesworth TD, Kollman C, Li Z, Brown AS, Close KL (2019) Validation of time in range as an outcome measure for diabetes clinical trials. Diabetes Care 42:400–405. 10.2337/dc18-1444 30352896 10.2337/dc18-1444 PMC6905478

[54] Bezerra MF, Neves C, Neves JS, Carvalho D (2023) Time in range and complications of diabetes: a cross-sectional analysis of patients with type 1 diabetes. Diabetol Metab Syndr 15:244. 10.1186/s13098-023-01219-2 38008747 10.1186/s13098-023-01219-2 PMC10680248

[55] Drucker DJ (2006) The biology of incretin hormones. Cell Metab 3:153–165. 10.1016/j.cmet.2006.01.004 16517403 10.1016/j.cmet.2006.01.004

[56] Yumuk V, Tsigos C, Fried M, Schindler K, Busetto L, Micic D, Toplak H (2015) European guidelines for obesity management in adults. Obes Facts 8:402–424. 10.1159/000442721 26641646 10.1159/000442721 PMC5644856

[57] Pozzilli P, Bosi E, Cirkel D, Harris J, Leech N, Tinahones FJ, Vantyghem MC, Vlasakakis G, Ziegler AG, Janmohamed S (2020) Randomized 52-Week Phase 2 Trial of Albiglutide Versus Placebo in Adult Patients With Newly Diagnosed Type 1 Diabetes. *J Clin Endocrinol Metab 105*: dgaa149. 10.1210/clinem/dgaa149. 10.1210/clinem/dgaa149 32219329

[58] European Medicines Agency Eperzan: European Public Assessment Report (EPAR) Available online: https://www.ema.europa.eu/en/medicines/human/EPAR/eperzan (accessed on 19 May 2025).

