# Supplementary material for: Glucagon-like peptide-1 receptor agonists as add-on therapy to insulin for type 1 diabetes mellitus: a systematic review and meta-analysis
Source: Hormones (Athens). 2025 Aug 4;24(4):1141–51. doi: 10.1007/s42000-025-00704-9 (PMC12678461; doi:10.1007/s42000-025-00704-9)
Supplement: Supplementary file 1 — Supplementary file1 (DOCX 292 KB) [file 42000_2025_704_MOESM1_ESM.docx]

**Supplementary Material**

**Glucagon-like peptide-1 receptor agonists as add-on therapy to insulin for type 1 diabetes mellitus: a systematic review and meta-analysis**

Eleni Rebelos^1,2^, Ioanna A. Anastasiou^1^, Nikolaos Tentolouris^1^, Thomas Karagiannis^3,4^, Apostolos Tsapas^3,4^, Ele Ferrannini^5^, Aris Liakos^3,4^

^1^ First Department of Propaedeutic Internal Medicine and Diabetes Center, School of Medicine, National and Kapodistrian University of Athens, Laiko General Hospital, Athens, Greece

^2^ Department of Clinical and Experimental Medicine, University of Pisa, 56126 Pisa, Italy

^3^ Clinical Research and Evidence-Based Medicine Unit, Second Medical Department, Aristotle University of Thessaloniki, Thessaloniki, Greece.

^4^ Diabetes Centre, Second Medical Department, Aristotle University of Thessaloniki, Konstantinoupoleos 49, 54642, Thessaloniki, Greece

^5^ CNR, Institute of Clinical Physiology, Pisa, Italy

**1.1 Study Characteristics**

1.1.1 Type of studies included

- Inclusion criteria

Randomised controlled trials (RCTs) with parallel or cross-over design and treatment duration of at least 12 weeks for meta-analysis of HbA_1c_ and body weight and at least 4 weeks (for all the other outcomes of interest), assessing the effect of any glucagon-like peptide 1 (GLP-1) analogue (GLP-1RA) currently in use (i.e. liraglutide, exenatide, Lixisenatide, dulaglutide, and semaglutide) as add-on to insulin therapy in patients with type 1 diabetes mellitus (T1D). Comparator addition of placebo or of no other drug to insulin therapy.

- Exclusion criteria

Non randomised clinical trials, and every other type of study.

Type of participants

- Inclusion criteria

Patients with T1D (no age limits), and irrespective of disease duration.

- Exclusion criteria

Patients with type 2 diabetes (T2D), or studies where a mixture of T1D and T2D patients and that it was not possible to extract the data of T1D patients only.

1.1.3. Type of interventions

- Inclusion Criteria

Eligible interventions included any GLP-1RA currently in use, without the addition of any other antidiabetic agent, given as add-on to insulin therapy in adults with type 1 diabetes.

- Exclusion Criteria

We excluded studies that assessed other antidiabetic agents as add-on to insulin, with or without the use of GLP-1 agonists.

1.1.4 Outcomes of interest

1. Change from baseline in HbA_1c_ (%)
2. Change from baseline in body weight (kg)
3. Change from baseline in total daily insulin dose (IU)
4. Change from baseline in time-in-range (%)
5. Change in plasma C-peptide levels
6. Change in plasma glucagon levels
7. Incidence of severe hypoglycemia

**1.2 Search strategies**

We searched Medline and Cochrane Central Register of Controlled Trials. We also searched for abstracts presented at the two major diabetes congresses (ADA and EASD) during the last 5 years (2019-2023).

Search date: 02 November 2023.

Yielded total references: 1796.

The exact keyword searches is presented below.

**Medline search strategy**

1. Diabetes Mellitus, Type 1
2. IDDM or T1DM or T1D
3. diabetes mellitus insulin dependent
4. juvenile onset diabetes
5. childhood diabetes
6. early onset diabetes
7. 1 or 2 or 3
8. Diabetes Insipidus
9. 4 not 5
10. glucagon-like peptide 1 receptor agonist
11. GLP-1 receptor agonist
12. GLP-1 RA
13. liraglutide
14. NN-2211
15. Victoza
16. lixisenatide
17. Adlyxin
18. AVE-010
19. dulaglutide
20. LY2189265
21. LY-2189265
22. Trulicity
23. exenatide
24. Bydureon
25. Byetta
26. AC-2993
27. semaglutide
28. NN9535
29. Ozempic
30. Rybelsus
31. #10 OR #11 OR #12 OR #13 OR #14 OR #15 OR #16 OR #17 OR #18 OR #19 OR #20 OR #21 OR #22 OR #23 OR #24 OR #25 OR #26 OR #27 OR #28 OR #29 OR #30
32. #9 AND #31
33. animals NOT humans
34. #32 NOT #33

Search: **#32 NOT #33** Sort by: **Most Recent**

((("diabetes mellitus, type 1"[MeSH Terms] OR "type 1 diabetes mellitus"[All Fields] OR "diabetes mellitus type 1"[All Fields] OR ("diabetes mellitus, type 1"[MeSH Terms] OR "type 1 diabetes mellitus"[All Fields] OR "iddm"[All Fields] OR "T1DM"[All Fields] OR "T1D"[All Fields]) OR ("diabetes mellitus, type 1"[MeSH Terms] OR "type 1 diabetes mellitus"[All Fields] OR ("diabetes"[All Fields] AND "mellitus"[All Fields] AND "insulin"[All Fields] AND "dependent"[All Fields]) OR "diabetes mellitus insulin dependent"[All Fields]) OR ("diabetes mellitus, type 1"[MeSH Terms] OR "type 1 diabetes mellitus"[All Fields] OR ("juvenile"[All Fields] AND "onset"[All Fields] AND "diabetes"[All Fields]) OR "juvenile onset diabetes"[All Fields]) OR (("childhood"[All Fields] OR "childhoods"[All Fields]) AND ("diabete"[All Fields] OR "diabetes mellitus"[MeSH Terms] OR ("diabetes"[All Fields] AND "mellitus"[All Fields]) OR "diabetes mellitus"[All Fields] OR "diabetes"[All Fields] OR "diabetes insipidus"[MeSH Terms] OR ("diabetes"[All Fields] AND "insipidus"[All Fields]) OR "diabetes insipidus"[All Fields] OR "diabetic"[All Fields] OR "diabetics"[All Fields] OR "diabets"[All Fields])) OR ("early"[All Fields] AND ("age of onset"[MeSH Terms] OR ("age"[All Fields] AND "onset"[All Fields]) OR "age of onset"[All Fields] OR "onset"[All Fields] OR "onsets"[All Fields] OR "onsetting"[All Fields]) AND ("diabete"[All Fields] OR "diabetes mellitus"[MeSH Terms] OR ("diabetes"[All Fields] AND "mellitus"[All Fields]) OR "diabetes mellitus"[All Fields] OR "diabetes"[All Fields] OR "diabetes insipidus"[MeSH Terms] OR ("diabetes"[All Fields] AND "insipidus"[All Fields]) OR "diabetes insipidus"[All Fields] OR "diabetic"[All Fields] OR "diabetics"[All Fields] OR "diabets"[All Fields]))) NOT ("diabetes insipidus"[MeSH Terms] OR ("diabetes"[All Fields] AND "insipidus"[All Fields]) OR "diabetes insipidus"[All Fields])) AND ((("glucagon like peptide 1 receptor"[MeSH Terms] OR ("glucagon like"[All Fields] AND "peptide 1"[All Fields] AND "receptor"[All Fields]) OR "glucagon like peptide 1 receptor"[All Fields] OR "glucagon like peptide 1 receptor"[All Fields]) AND ("agonist"[All Fields] OR "agonist s"[All Fields] OR "agonistic"[All Fields] OR "agonistically"[All Fields] OR "agonistics"[All Fields] OR "agonists"[MeSH Subheading] OR "agonists"[All Fields])) OR (("glucagon like peptide 1 receptor"[MeSH Terms] OR ("glucagon like"[All Fields] AND "peptide 1"[All Fields] AND "receptor"[All Fields]) OR "glucagon like peptide 1 receptor"[All Fields] OR "glp 1 receptor"[All Fields]) AND ("agonist"[All Fields] OR "agonist s"[All Fields] OR "agonistic"[All Fields] OR "agonistically"[All Fields] OR "agonistics"[All Fields] OR "agonists"[MeSH Subheading] OR "agonists"[All Fields])) OR (("glucagon like peptide 1"[MeSH Terms] OR "glucagon like peptide 1"[All Fields] OR "glp 1"[All Fields]) AND "RA"[All Fields]) OR ("liraglutid"[All Fields] OR "liraglutide"[MeSH Terms] OR "liraglutide"[All Fields] OR "liraglutide s"[All Fields]) OR ("liraglutide"[MeSH Terms] OR "liraglutide"[All Fields] OR "nn 2211"[All Fields]) OR ("liraglutid"[All Fields] OR "liraglutide"[MeSH Terms] OR "liraglutide"[All Fields] OR "victoza"[All Fields] OR "liraglutide s"[All Fields]) OR ("lixisenatide"[Supplementary Concept] OR "lixisenatide"[All Fields]) OR ("lixisenatide"[Supplementary Concept] OR "lixisenatide"[All Fields] OR "adlyxin"[All Fields]) OR ("lixisenatide"[Supplementary Concept] OR "lixisenatide"[All Fields]) OR ("dulaglutide"[Supplementary Concept] OR "dulaglutide"[All Fields]) OR ("dulaglutide"[Supplementary Concept] OR "dulaglutide"[All Fields] OR "ly2189265"[All Fields]) OR ("dulaglutide"[Supplementary Concept] OR "dulaglutide"[All Fields] OR "ly 2189265"[All Fields]) OR ("dulaglutide"[Supplementary Concept] OR "dulaglutide"[All Fields] OR "trulicity"[All Fields]) OR ("exenatide"[MeSH Terms] OR "exenatide"[All Fields] OR "exenatide s"[All Fields]) OR ("exenatide"[MeSH Terms] OR "exenatide"[All Fields] OR "bydureon"[All Fields] OR "exenatide s"[All Fields]) OR ("exenatide"[MeSH Terms] OR "exenatide"[All Fields] OR "byetta"[All Fields] OR "exenatide s"[All Fields]) OR ("exenatide"[MeSH Terms] OR "exenatide"[All Fields] OR "ac 2993"[All Fields]) OR ("semaglutide"[Supplementary Concept] OR "semaglutide"[All Fields]) OR "NN9535"[All Fields] OR ("semaglutide"[Supplementary Concept] OR "semaglutide"[All Fields] OR "ozempic"[All Fields]) OR ("semaglutide"[Supplementary Concept] OR "semaglutide"[All Fields] OR "rybelsus"[All Fields]))) NOT (("animals"[MeSH Terms:noexp] OR "animals"[All Fields]) NOT ("human s"[All Fields] OR "humans"[MeSH Terms] OR "humans"[All Fields] OR "human"[All Fields]))

**Translations**

**Diabetes Mellitus, Type 1:** "diabetes mellitus, type 1"[MeSH Terms] OR "type 1 diabetes mellitus"[All Fields] OR "diabetes mellitus type 1"[All Fields]

**IDDM:** "diabetes mellitus, type 1"[MeSH Terms] OR "type 1 diabetes mellitus"[All Fields] OR "iddm"[All Fields]

**diabetes mellitus insulin dependent:** "diabetes mellitus, type 1"[MeSH Terms] OR "type 1 diabetes mellitus"[All Fields] OR ("diabetes"[All Fields] AND "mellitus"[All Fields] AND "insulin"[All Fields] AND "dependent"[All Fields]) OR "diabetes mellitus insulin dependent"[All Fields]

**juvenile onset diabetes:** "diabetes mellitus, type 1"[MeSH Terms] OR "type 1 diabetes mellitus"[All Fields] OR ("juvenile"[All Fields] AND "onset"[All Fields] AND "diabetes"[All Fields]) OR "juvenile onset diabetes"[All Fields]

**childhood:** "childhood"[All Fields] OR "childhoods"[All Fields]

**diabetes:** "diabete"[All Fields] OR "diabetes mellitus"[MeSH Terms] OR ("diabetes"[All Fields] AND "mellitus"[All Fields]) OR "diabetes mellitus"[All Fields] OR "diabetes"[All Fields] OR "diabetes insipidus"[MeSH Terms] OR ("diabetes"[All Fields] AND "insipidus"[All Fields]) OR "diabetes insipidus"[All Fields] OR "diabetic"[All Fields] OR "diabetics"[All Fields] OR "diabets"[All Fields]

**onset:** "age of onset"[MeSH Terms] OR ("age"[All Fields] AND "onset"[All Fields]) OR "age of onset"[All Fields] OR "onset"[All Fields] OR "onsets"[All Fields] OR "onsetting"[All Fields]

**diabetes:** "diabete"[All Fields] OR "diabetes mellitus"[MeSH Terms] OR ("diabetes"[All Fields] AND "mellitus"[All Fields]) OR "diabetes mellitus"[All Fields] OR "diabetes"[All Fields] OR "diabetes insipidus"[MeSH Terms] OR ("diabetes"[All Fields] AND "insipidus"[All Fields]) OR "diabetes insipidus"[All Fields] OR "diabetic"[All Fields] OR "diabetics"[All Fields] OR "diabets"[All Fields]

**diabetes insipidus:** "diabetes insipidus"[MeSH Terms] OR ("diabetes"[All Fields] AND "insipidus"[All Fields]) OR "diabetes insipidus"[All Fields]

**glucagon-like peptide 1 receptor:** "glucagon-like peptide-1 receptor"[MeSH Terms] OR ("glucagon-like"[All Fields] AND "peptide-1"[All Fields] AND "receptor"[All Fields]) OR "glucagon-like peptide-1 receptor"[All Fields] OR "glucagon like peptide 1 receptor"[All Fields]

**agonist:** "agonist"[All Fields] OR "agonist's"[All Fields] OR "agonistic"[All Fields] OR "agonistically"[All Fields] OR "agonistics"[All Fields] OR "agonists"[Subheading] OR "agonists"[All Fields]

**GLP-1 receptor:** "glucagon-like peptide-1 receptor"[MeSH Terms] OR ("glucagon-like"[All Fields] AND "peptide-1"[All Fields] AND "receptor"[All Fields]) OR "glucagon-like peptide-1 receptor"[All Fields] OR "glp 1 receptor"[All Fields]

**agonist:** "agonist"[All Fields] OR "agonist's"[All Fields] OR "agonistic"[All Fields] OR "agonistically"[All Fields] OR "agonistics"[All Fields] OR "agonists"[Subheading] OR "agonists"[All Fields]

**GLP-1:** "glucagon-like peptide 1"[MeSH Terms] OR "glucagon-like peptide 1"[All Fields] OR "glp 1"[All Fields]

**liraglutide:** "liraglutid"[All Fields] OR "liraglutide"[MeSH Terms] OR "liraglutide"[All Fields] OR "liraglutide's"[All Fields]

**NN-2211:** "liraglutide"[MeSH Terms] OR "liraglutide"[All Fields] OR "nn 2211"[All Fields]

**Victoza:** "liraglutid"[All Fields] OR "liraglutide"[MeSH Terms] OR "liraglutide"[All Fields] OR "victoza"[All Fields] OR "liraglutide's"[All Fields]

**lixisenatide:** "lixisenatide"[Supplementary Concept] OR "lixisenatide"[All Fields]

**Adlyxin:** "lixisenatide"[Supplementary Concept] OR "lixisenatide"[All Fields] OR "adlyxin"[All Fields]

**AVE-010:** "lixisenatide"[Supplementary Concept] OR "lixisenatide"[All Fields] OR "ave 010"[All Fields]

**dulaglutide:** "dulaglutide"[Supplementary Concept] OR "dulaglutide"[All Fields]

**LY2189265:** "dulaglutide"[Supplementary Concept] OR "dulaglutide"[All Fields] OR "ly2189265"[All Fields]

**LY-2189265:** "dulaglutide"[Supplementary Concept] OR "dulaglutide"[All Fields] OR "ly 2189265"[All Fields]

**Trulicity:** "dulaglutide"[Supplementary Concept] OR "dulaglutide"[All Fields] OR "trulicity"[All Fields]

**exenatide:** "exenatide"[MeSH Terms] OR "exenatide"[All Fields] OR "exenatide's"[All Fields]

**Bydureon:** "exenatide"[MeSH Terms] OR "exenatide"[All Fields] OR "bydureon"[All Fields] OR "exenatide's"[All Fields]

**Byetta:** "exenatide"[MeSH Terms] OR "exenatide"[All Fields] OR "byetta"[All Fields] OR "exenatide's"[All Fields]

**AC-2993:** "exenatide"[MeSH Terms] OR "exenatide"[All Fields] OR "ac 2993"[All Fields]

**semaglutide:** "semaglutide"[Supplementary Concept] OR "semaglutide"[All Fields]

**Ozempic:** "semaglutide"[Supplementary Concept] OR "semaglutide"[All Fields] OR "ozempic"[All Fields]

**Rybelsus:** "semaglutide"[Supplementary Concept] OR "semaglutide"[All Fields] OR "rybelsus"[All Fields]

**Animals:** "animals"[MeSH Terms:noexp] OR animals[All Fields]

**humans:** "human's"[All Fields] OR "humans"[MeSH Terms] OR "humans"[All Fields] OR "human"[All Fields]

**Cochrane : 941** records

EASD 2019-2023: 5 records

ADA: 2019-2023: 6 records

We then used the Systematic Review Accelerator (<https://sr-accelerator.com>) to remove duplicates.

**1.3. Selection of Studies**

Records retrieved from database searches were imported in the online systematic literature review software Systematic Review Accelerator (<https://sr-accelerator.com>). After removing duplicates, two independent reviewers (ER and IA) screened titles and abstracts. Any disagreements were resolved by a third independent reviewer. Subsequently, the same pair of reviewers independently screened the full-text of potentially eligible articles, according to the meta-analysis inclusion criteria. Any discrepancies were again resolved by a third independent reviewer (AL).

**2. Calculations needed during the data extraction process**

**2.1 HbA_1c_ (%)**

Ahren et al. reported estimated treatment difference (ETD) for all liraglutide doses versus placebo [1]. As this study had 3 treatment arms and a placebo (PBO) arm, we used the formula SQRT ((4+1)/2)= SQRT(2.5) = 1.58, to adjust SE. SE of each comparison to placebo, was thus multiplied by 1.58 [2].

Brock et al. reported baseline and EOT median [IQR] values for HbA_1c_ in mmol/mol [3]. We first transformed HbA_1c_ values in %. Median [IQR] were transformed in mean and SD based on the method by Wan et al [4]. SD of the change was then calculated from baseline SD and final SD as described in the Cochrane Handbook guidelines (section 6.5.2.8) [2]. The correlation coefficient was calculated from the study by Dejgaard 2020 et al.

Dandona et al. reported placebo adjusted change of HbA_1c_ [5]. This information was directly used in the Revman.

Dejgaard 2016 et al. reported ETD [6], this data was directly used in Revman.

Dejgaard 2019 et al. reported HbA_1c_ in mmol/mol as median (95% CI) [7]. We first transformed the data in %. SD of the change was borrowed by a similar study from the same group (Dejgaard 2016).

Dejgaard 2020 et al. reported ETD for HbA_1c_ in % [8]. This data was directly used in Revman.

Dube et al. reported baseline and EOT HbA_1c_ values as mean ± SEM [9]. SD of the change was borrowed by a similar study.

Frandsen et al. reported change from baseline for Liraglutide and PBO [10]. This data was directly used in Revman.

Ghanim et al. reported change in HbA_1c_ for both groups at Figure 1A [11]. The data were extracted with Plotdigitizer.

Hamamoto et al. reported baseline and EOT HbA_1c_ in % [12]. Imputation for change SD was needed from baseline and final SD.

Herold et al. reported the group differences between exenatide and PBO [13]. This data was directly used in Revman.

Johansen et al. reported ETD of HbA_1c_ [14]. This data was directly used in Revman.

Kuhadiya et al. reported change of HbA_1c_ for placebo and each treatment arm [15]. As this study had 3 treatment arms and a PBO arm, we used the formula SQRT ((4+1)/2)= SQRT(2.5) = 1.58, to adjust SE. SE of each comparison to placebo, was thus multiplied by 1.58.

Kumar et al. reported HbA_1c_ values at baseline and EOT [16]. Imputation was used to calculate SE of the change.

Mathieu et al. reported ETD of HbA_1c_ [17]. As this study had 3 treatment arms and a PBO arm, we used the formula SQRT ((4+1)/2)= SQRT(2.5) = 1.58, to adjust SE. SE of each comparison to placebo, was thus multiplied by 1.58.

Navodnik et al. reported change of HbA_1c_ for Semaglutide and PBO [18]. This data was directly used in Revman.

Sarkar et al. reported HbA_1c_ on and off exenatide [19]. Imputation was used to calculate SE of the change.

Thivolet et al. reported HbA_1c_ in median [95% CI] [20]. Transformation was first used to calculate mean and SD. Imputation was used to calculate SE of the change.

Von Herrath et al. reported ETD [21]. This data was directly used in Revman.

Zenz et al. reported differences between baseline and EOT as median (min, max) [22]. Transformation was first used to calculate mean and SD based on Wan et al. [4].

**2.2 Time-in-range (%)**

Dejgaard et al. (2016) used blinded CGM for 6 consecutive days at baseline and EOT and reported the between-group differences in TIR in hours per day as means and 95% CI [6]. We calculated TIR (%) by multiplying these values by 100 and dividing by 24.

Dejgaard et al. (2020) used blinded CGM for 6 consecutive days and reported the between-group differences in TIR in % per day as means and 95% CI [8] and thus the data were used directly in RevMan.

Dube et al. used a more stringent blood glucose control to define TIR (3.8-7.8 mmol/L) [9]. Participants used the CGM for 3 to 7 days. There was no mention if CGM was blinded in this study. They reported TIR outcomes in % at baseline and EOT. As SD of the change was not given in this study the average of the SD at baseline and EOT was used.

Frandsen et al. used blinded CGM for 4 days at baseline and EOT [10]. They reported changes in TIR for each group separately in h/day. We calculated TIR (%) by multiplying these values by 100 and dividing by 24.

Ghanim et al. used blinded CGM for 2 weeks and a more stringent glycemic control between 3.89–8.89 mmol/L to define TIR [11]. TIR data were given in % per day and thus the data were used directly in RevMan.

Jiang et al. used for 3 days [23]. There was no mention if CGM was blinded in this study. They reported the CGM outcomes at baseline and EOT for the two groups separately as percentage of time below 3.9 mmol/L (PT1) and percentage of time above 10 mmol/L (PT3). We thus subtracted PT1 and PT3 from 100 to calculate TIR. As SD of the change in TIR was not given in this study, SD was imputed.

Johansen et al. used blinded CGM for 6 consecutive days [14]. They reported % TIR change between the two intervention as ETD. The data were directly used in Revman.

Kielgast et al. used blinded CGM for 3 days [24]. Baseline and EOT TIR data were given as hours/day. We calculated TIR (%) by multiplying these values by 100 and dividing by 24. As only baseline and EOT mean and SD were given, SD of the change was assumed to be similar as the mean SD of the baseline and EOT.

Kuhadiya et al. used blinded CGM for 12 weeks – i.e. the entire study duration [15]. They used a more stringent blood glucose control to define TIR (70-160 mg/dl, 3.8-8.8 mmol/L). Changed over 12 weeks in TIR in % was given in table 2. This data was used in Revman.

Navodnik et al. used CGM for the entire study duration [18]. There was no mention if CGM were blinded in this study. TIR was defined as 3.9-10 mmol/L. Change in TIR (%) was given in Table 2. This information was directly used in Revman.

Pieber et al. reported the CCM methods and results in the Supplement [25]. Blinded CGM was used for 3-6 days at baseline and at the EOT. Duration of low interstitial glucose (IG) ((≤3.9 mmol/L) (h) and duration of high IG (≥10.0 mmol/L) (h) were given as ETD and 95% CI. As time-in-range was not directly reported in this study, duration of normal IG was first calculating by subtracting low and high IG from 24 to calculate TIR in h. TIR in % was then calculated by multiplying by 100 and dividing by 24. As it was not possible to estimate SD of the change from this data, it was imputed from a similar study [8].

**2.3 Body weight (Kg)**

Ahren et al. reported change in body in Figure 1C [1]. We used Plotdigitizer to extract the data. As this study had 3 treatment arms and a PBO arm, we used the formula SQRT ((4+1)/2)= SQRT(2.5) = 1.58, to adjust SE. SE of each comparison to placebo, was thus multiplied by 1.58.

Brock et al. reported the weight reduction with Liraglutide compared to placebo [3]. This data was used in Revman.

Dandona et al. reported placebo adjusted change of body weight [5]. This information was directly used in the Revman.

Dejgaard et al. (2016) reported ETD [6], this data was directly used in Revman.

Dejgaard et al. (2019) reported on BMI data [7], thus this study was not used in the meta-analysis.

Dejgaard et al. (2020) reported ETD [8]. This data was directly used in Revman.

Dube et al. reported baseline and EOT BW values as means ± SEM [9]. SD of the change was imputed.

Frandsen et al. reported change from baseline for Liraglutide and PBO [10]. This data was directly used in Revman.

Ghanim et al. reported change in BW for both groups at Figure 1A [11]. The data were extracted with Plotdigitizer.

Hamamoto et al. did not report body weight [12], thus this study was not used in the meta-analysis.

Herold et al. reported the group differences between exenatide and PBO [13]. This data was directly used in Revman.

Johansen et al. reported ETD of body weight [14]. This data was directly used in Revman.

Kuhadiya et al. reported change of body weight for placebo and each treatment arm [15]. As this study had 3 treatment arms and a PBO arm, we used the formula SQRT ((4+1)/2)= SQRT(2.5) = 1.58, to adjust SE. SE of each comparison to placebo, was thus multiplied by 1.58.

Kumar et al. reported body weight values at baseline and EOT[16]. Imputation was used to calculate SE of the change.

Mathieu et al. reported ETD of BW [17]. As this study had 3 treatment arms and a PBO arm, we used the formula SQRT ((4+1)/2)= SQRT(2.5) = 1.58, to adjust SE. SE of each comparison to placebo, was thus multiplied by 1.58.

Navodnik et al. reported change of BW for Semaglutide and PBO [18]. This data was directly used in Revman.

Sarkar et al. reported body weight on and off exenatide [19]. Imputation was used to calculate SE of the change.

Thivolet et al. reported change in BW with PBO and Dulaglutide in median [95% CI] [20]. Transformation was first used to calculate mean and SD based on Wan et al.

Von Herrath et al. reported ETD [21]. This data was directly used in Revman.

Zenz et al. reported differences between baseline and EOT as median (min, max) [22]. Transformation was first used to calculate mean and SD based on Wan et al. [4].

**2.4 TID (U/day)**

Ahren et al. reported TID at EOT as ratio to the baseline values (in Figure 1B) [1]. We used PlotDigitizer to export the data. As it was not possible to obtain an SD of the change for this study (and this was one of the biggest studies included in this meta-analysis), we assumed that SE of the change in TID was equal to SE in TID at the EOT. As this study had 3 treatment arms and a PBO arm, we used the formula SQRT ((4+1)/2)= SQRT(2.5) = 1.58, to adjust SE. SE of each comparison to placebo, was thus multiplied by 1.58.

Ballav et al. reported change in TID (U/day) in Figure 3B and also in the Supplement [26]. We used the information given in the supplement directly in Revman.

In the study by Brock et al. TID at baseline and EOT are given [3]. Estimates of test for treatment effect are in % or absolute values (95% confidence interval) (Table 3). We calculated the deltas for each group based on the data in Table 3. SE was borrowed from the study by Dejgaard et al. 2020 (2.9592).

Dube et al. reported total insulin dose (U/day) at baseline and end-of-treatment in Table 1[9]. As SD of the change was not given, it was taken from a relatively similar study (Dejgaard et al. LiraPump 2020, N= 44).

Frandsen et al. did not report SD of the change [10]; thus SE was borrowed from a similar study.

Ghanim et al. reported TID in U/Kg [11]. As body weight at baseline and end of treatment was also given, TID (U/day) was calculated for both study arms. SD of the difference was not given. Thus, SE was borrowed from a similar study.

Hamamoto et al. did not report SD of the change [12]; SE was borrowed from a similar study.

Herold et al. did not give SD of the change [13]; SE was borrowed from a similar study.

Jiang et al. did not report SD of the change [23]. SE was borrowed from a similar study.

Kielgast et al. reported TID in U/kg [24]; this study was not included in the meta-analysis for TID (U/day).

Kuhadiya et al. reported TID (U/day) in Table 2 as change for each arm over treatment [15]. Mean and SEM are given. As this study had 4 arms in total, we used the formula SQRT ((4+1)/2)= SQRT(2.5) = 1.58, to adjust SE. The individual SE for each comparison to placebo, were thus multiplied by 1.58.

Pieber et al. gave the results for TID in Supplemental Table S2 [25].

Sarkar et al. reported TID data as U/Kg [19]. As body weight on and off exenatide were given, TID (U/day) on and off exenatide were calculated and these values were then used to calculate MD. SD was borrowed from a similar study.

Thivolet et al. reported TID in U/Kg [20]. As both baseline TID and body weight (BW) and change in TID and BW were given we calculated mean differences. SD of the change was borrowed from a similar study.

In the study by Van Meijel et al. baseline TID (U/d) is given in Table 1 [27]. Basal and boluses ID during exenatide and during placebo are also given in the main text. Based on this information we calculated deltas of TID. SD of the change was borrowed from a similar study.

Zenz et al. gave the differences between baseline and 3-month treatment in Table 1, as minimum, median and maximum [22]. We used the equations described by Wan et al. [4] to calculate mean and SD of the change.

Mathieu et al. reported TID (U/day) in Figure 1B and in the main text as ETR [17]. We used Plotdigitizer to extract the EOT mean values of TID and calculated mean changes by subtracting these values from the baseline information given in Table 1. As this was the largest study in the meta-analysis, it was assumed that SD at EOT was the same as SD of the change.

Von Herrath et al. reported baseline mean TDI (U/Kg) 0.30 in the Liraglutide group and 0.32 in the PBO group [21]. BW at Lira was 74.0 Kg and 72.8 Kg for PBO. As in the supplement the MD of BW was given, BW post was calculated. The change in TID in U/Kg was extracted using Plotdigitizer (**Figure S2A**). SE of the change was borrowed from a similar study.

**2.5 Total insulin dose (U/kg)**

For the study by Ballav et al. change in TID (U/kg) was calculated by dividing the change in TID by EOT BW [26]. SE of the change was borrowed from a similar study.

For Brock et al. the information given in Table 3 was used to calculate TID (U/kg) [3]. SE was borrowed from a similar study.

Dejgaard et al. (2016) was giving TID (U/kg) in Table 2 [6].

Dejgaard et al. (2019) reported only mean (95% CI) of baseline and EOT for TID (U/day). As only BMI (but not body weight) was given in this abstract, this study was not included in the TID pro Kg meta-analysis.

Dejgaard et al. (2020) reported TID in (U/day) [8]. TID (U/kg) was calculated by dividing the change and 95% CI by final body weight.

Dube et al. reported TID (U/day) as mean ± SEM [28]. TID (U/kg) at each time point was calculated by dividing TID by the respective BW; and deltas for Lira and PBO treatment were calculated. SE was borrowed from a similar study.

Johansen et al. reported TID in (U/Kg) in Table 2 [14].

Kuhadiya et al. reported the change in TID (U/ day) in mean±SEM [15]. The change in TID (U/kg) was calculated by dividing this change by EOT BW for each group. SE was borrowed from a similar study.

Frandsen et al. gave bolus insulin as U/day and U/Kg, whereas basal insulin dose was given as U/day at baseline and EOT [10]. Using the corresponding body weights, MD of TID (U/kg) was calculated. SE was borrowed from a similar study.

Ghanim et al. reported TID pro Kg as mean±SEM at baseline and EOT [11]. These data were used to calculate MD. SE was borrowed from a similar study.

The study by Hamamoto et al. was excluded as TID was given in U/day and body weight was not reported [12].

Herold et al. gave information regarding TID pro Kg in Figure 3 [13]. SE for the MD was borrowed from a similar study.

Jiang et al. reported TID as mean SD at baseline and EOT [23]. TID (U/kg) was calculated based on these information. SE was borrowed from a similar study.

Kumar et al. reported TID (U/kg) as mean (SD) (Table 2) [16]. SE was borrowed from a similar study.

Navodnik reported the change in TID (U/day) as mean (SD) (and min max) [18]. We calculated TID pro Kg dividing by EOT BW. SE was borrowed a similar study.

In the study by Sarkar et al. SE of the change in TID is not given [19]. It was borrowed from a similar study.

Van Meijel et al. reported separately basal and bolus insulin doses after exenatide treatment and after placebo in U/day [27]. TID (U/day) were first calculated and using the BW of each period mean TID (U/kg) were calculated.

Zenz et al. gave the differences between baseline and 3-month treatment in Table 1, as minimun, median and maximum [22]. We used the equations described by Wan et al. to calculate mean of the change in TID (U/day) and BW. These values were then used to calculate the change in TID (U/kg).

Pieber et al. reported in the supplemental table S2 TID as estimated treatment difference (ETD). These values were divided by EOT body weight to obtain estimated treatment difference (ETD), [95% confidence intervals] in TID (U/kg).

**3.0 Subgroup analysis based on baseline C-peptide levels**

Ahren et al. performed a subgroup analysis for C-peptide positive and C-peptide negative patients. The placebo-corrected data for these subjects were used [1]. As SE of the change was not given it was borrowed by the subgroup analysis of Mathieu et al. [17].

Brock et al. did not measure C-peptide levels; this study was excluded from the subgroup analysis [3].

Dandona et al. assessed patients who had no detectable C-peptide values in plasma [5].

Dejgaard et al. (2016) included patients with measurable C-peptide levels, but they did not provide a subgroup analysis; this study was excluded from the subgroup analysis [6].

Dejgaard et al. (2019) studied exclusively patients with newly diagnosed T1D and stimulated C-peptide ≥ 200 pmol/L [7].

Dejgaard et al. (2020) studied both patients with residual and no residual β-cell function. As a subgroup analysis was not provided in this study, it was excluded from the subgroup analysis [8].

In the study by Dube et al. C-peptide values were not measured. This study was excluded from the subgroup analysis [28].

Frandsen et al. studied exclusively patients with no residual b-cell function (defined as stimulated plasma C-peptide < 60 pmol/L) [10].

Ghanim et al. studied exclusively patients who had no detectable C-peptide values [11].

Hamamoto et al. studied patients who had residual insulin secretion, which was defined as C-peptide values > 0.3 ng/ml [12].

Herold et al. performed a subgroup analysis for C-peptide positive and C-peptide negative patients. The placebo-corrected data for these subjects were used [13].

Johansen et al. included both patients with preserved and not preserved C-peptide values, but a subgroup analysis was not given [14]. This study was excluded from the subgroup analysis.

Mathieu et al. performed a subgroup analysis for C-peptide positive and C-peptide negative patients. The placebo-corrected data for these subjects were used [17].

Kuhadiya et al. included patients who had fasting C-peptide <0.1 nmol/L [15].

Kumar et al. included patients with new onset of T1D [16]. It was assumed that these were C-peptide positive patients.

Mathieu et al. reported the outcomes in HbA_1c_ in C-peptide positive and C-peptide negative patients in Supplementary Figure S3 [17]. Data were extracted using Plotidigitizer.

Navodnik et al. did not measure C-peptide levels; this study was excluded from the subgroup analysis [18].

Sarkar et al. studied patients with long lasting T1D (mean diabetes duration 20.5 ± 11.8 years), C-peptide values were not measured [19]. This study was excluded from the subgroup analysis.

Thivolet et al. studied patients with T1DM with β-cell reserve [20].

Von Herrath et al. studied exclusively patients with recently diagnosed type 1 diabetes and residual β-cell function [21]**.**

Zenz et al. studied exclusively C-peptide positive patients [22].

**Supplementary Figure 1** – Subgroup analysis on the effect of GLP-1RA compared with placebo on HbA_1c_ (%) in patients with preserved *vs* not preserved C-peptide levels *.


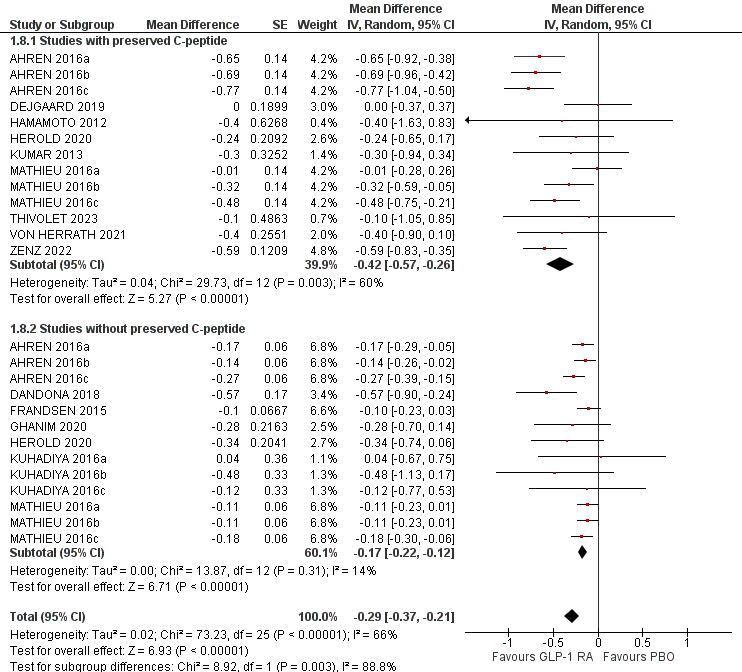


*a: liraglutide 0.6 mg, b: liraglutide 1.2 mg, c: liraglutide 1.8 mg. Preserved C-peptide levels were used as defined in each individual study.

**Supplementary** **Figure 2**- Funnel plot of the comparison GLP1-RA vs PBO, for HbA_1c_.


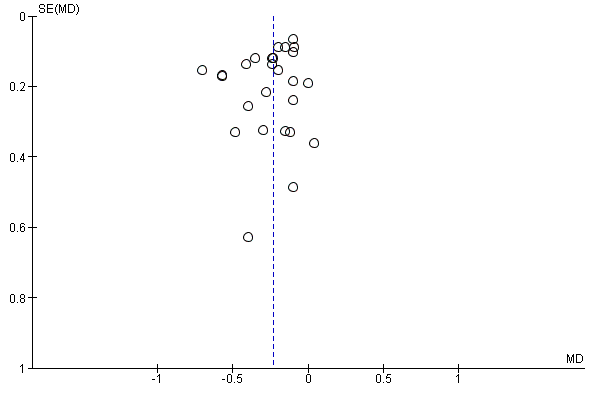


**Supplementary Figure 3**- Effect of GLP-1RA compared with placebo on total insulin dose (TID) per day (**A**), and per kg (**B**). *a: Liraglutide 0.6 mg, b: Liraglutide 1.2 mg, c: Liraglutide 1.8 mg.

**A**)


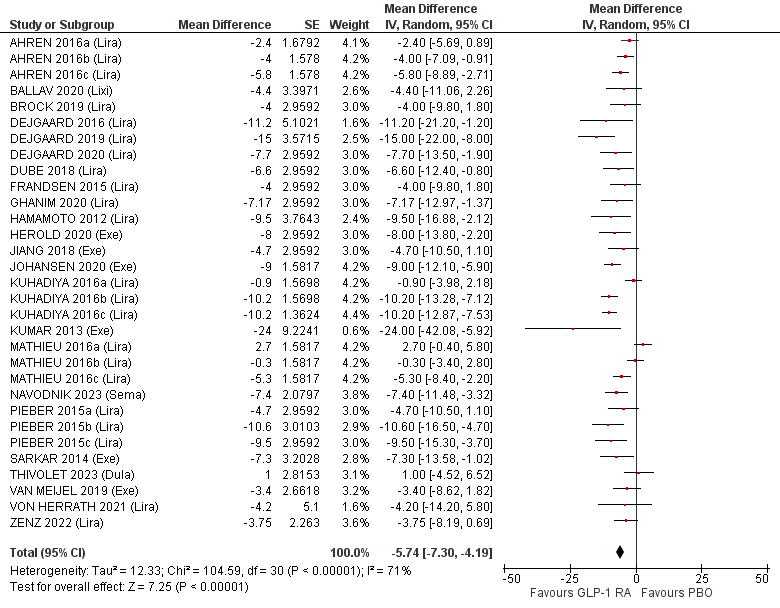


**B**)


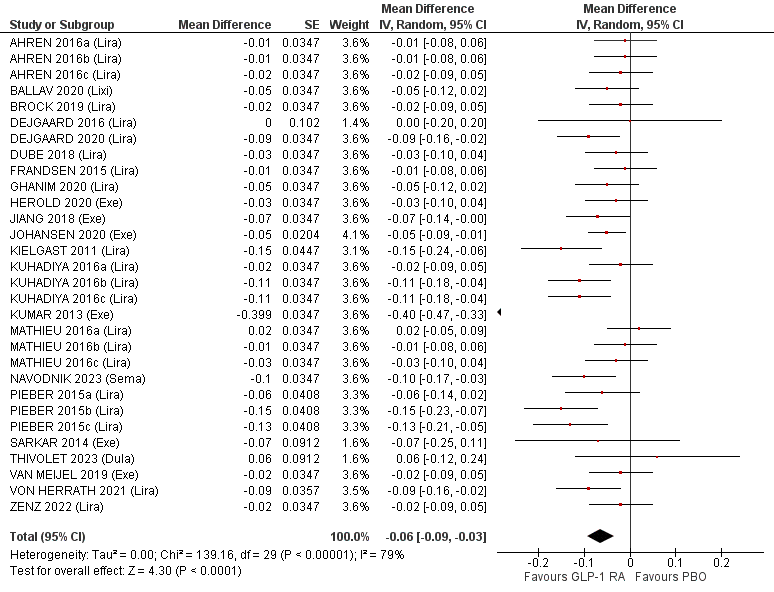


**Sensitivity analysis**

**As several studies did not report measures of dispersion, we performed a sensitivity analysis for the primary outcome, excluding studies in which measures of dispersion were imputed. The result is comparable to the original analysis (Supplementary Figure 4).**

**Supplementary Figure 4**


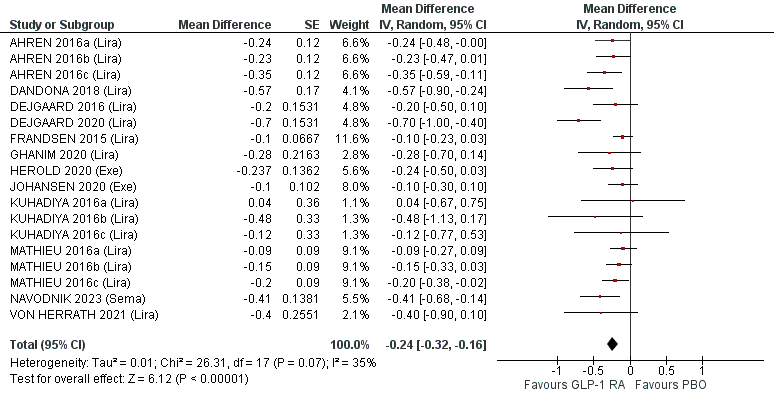


Risk of bias assessment for the primary outcome*.


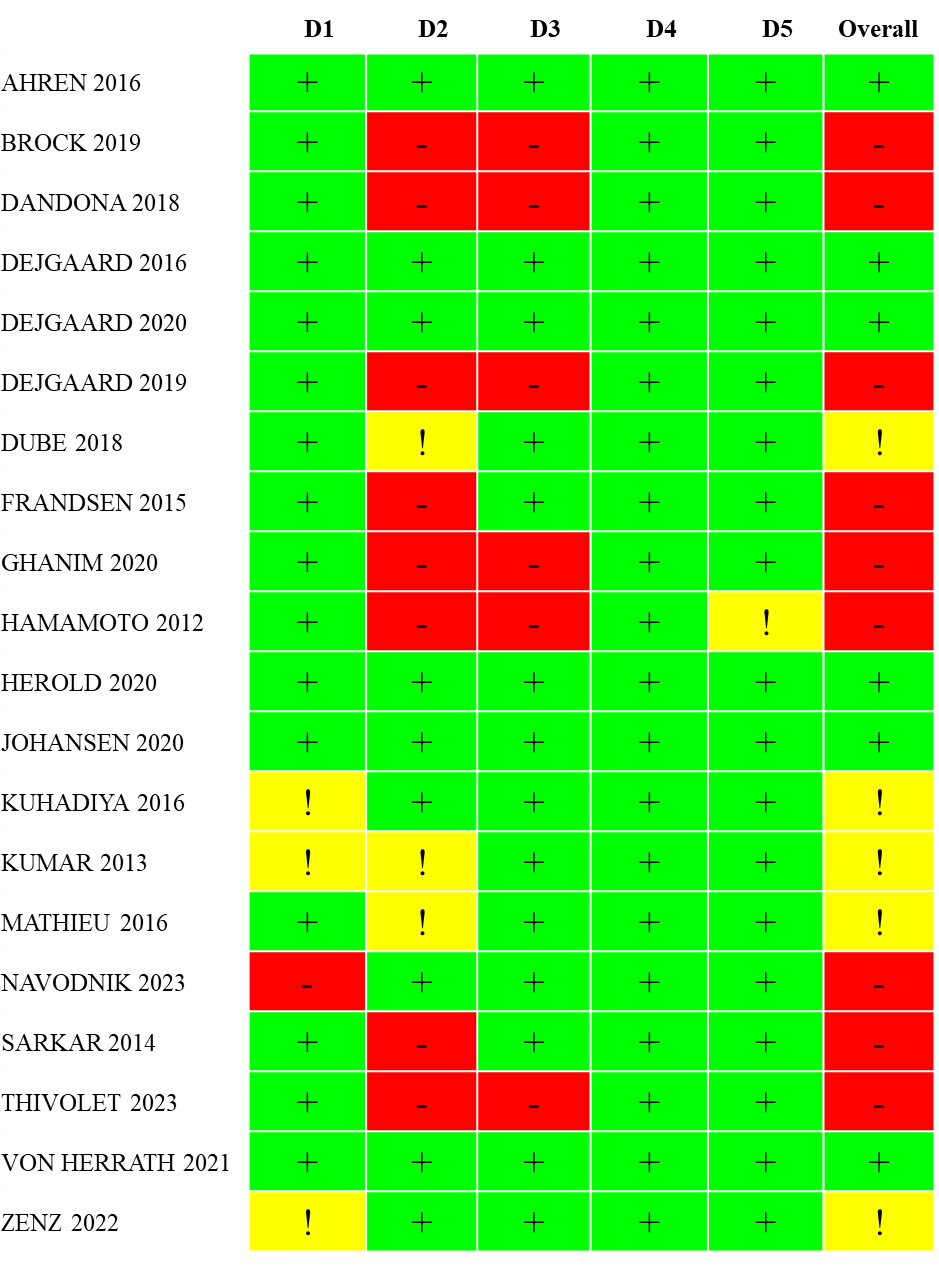


*D1: Domain 1. Randomization process; D2: Domain 2. Deviations from intended interventions; D3: Domain 3. Missing outcome data; D4: Domain 4. Measurement of the outcome; D5: Selection of the reported result.

Green colour indicates low risk of bias, red colour high risk of bias, and yellow indicates some concerns.

**Risk of bias assessment for studies not included in the primary outcome evaluation*.
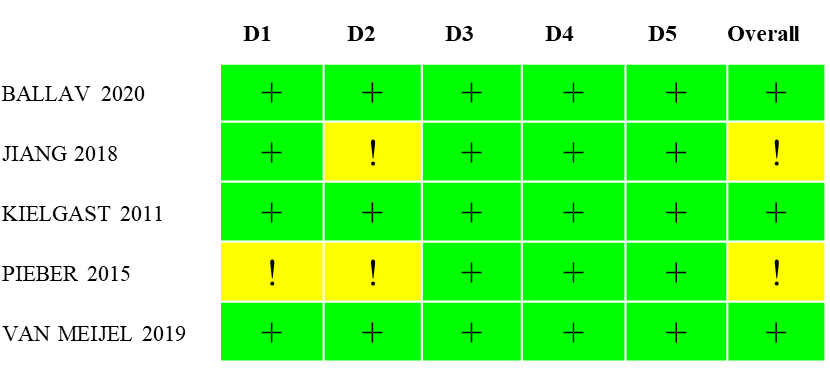
**

***D1: Domain 1. Randomization process; D2: Domain 2. Deviations from intended interventions; D3: Domain 3. Missing outcome data; D4: Domain 4. Measurement of the outcome; D5: Selection of the reported result.**

**Green colour indicates low risk of bias, red colour high risk of bias, and yellow indicates some concerns.**

# PRISMA 2020 Main Checklist

| **Topic** | **No.** | **Item** | **Location where item is reported** |
| --- | --- | --- | --- |
| **TITLE** |  |  |  |
| **Title** | 1 | Identify the report as a systematic review. | 1 |
| **ABSTRACT** |  |  |  |
| **Abstract** | 2 | See the PRISMA 2020 for Abstracts checklist |  |
| **INTRODUCTION** |  |  |  |
| **Rationale** | 3 | Describe the rationale for the review in the context of existing knowledge. | 3 |
| **Objectives** | 4 | Provide an explicit statement of the objective(s) or question(s) the review addresses. | 3, 4 |
| **METHODS** |  |  |  |
| **Eligibility criteria** | 5 | Specify the inclusion and exclusion criteria for the review and how studies were grouped for the syntheses. | 4 |
| **Information sources** | 6 | Specify all databases, registers, websites, organisations, reference lists and other sources searched or consulted to identify studies. Specify the date when each source was last searched or consulted. | 4 |
| **Search strategy** | 7 | Present the full search strategies for all databases, registers and websites, including any filters and limits used. | Supplement |
| **Selection process** | 8 | Specify the methods used to decide whether a study met the inclusion criteria of the review, including how many reviewers screened each record and each report retrieved, whether they worked independently, and if applicable, details of automation tools used in the process. | 4, 5 |
| **Data collection process** | 9 | Specify the methods used to collect data from reports, including how many reviewers collected data from each report, whether they worked independently, any processes for obtaining or confirming data from study investigators, and if applicable, details of automation tools used in the process. | 4, 5 |
| **Data items** | 10a | List and define all outcomes for which data were sought. Specify whether all results that were compatible with each outcome domain in each study were sought (e.g. for all measures, time points, analyses), and if not, the methods used to decide which results to collect. | 4, 5 |
|  | 10b | List and define all other variables for which data were sought (e.g. participant and intervention characteristics, funding sources). Describe any assumptions made about any missing or unclear information. | 5 |
| **Study risk of bias assessment** | 11 | Specify the methods used to assess risk of bias in the included studies, including details of the tool(s) used, how many reviewers assessed each study and whether they worked independently, and if applicable, details of automation tools used in the process. | 5 |
| **Effect measures** | 12 | Specify for each outcome the effect measure(s) (e.g. risk ratio, mean difference) used in the synthesis or presentation of results. | 5 |
| **Synthesis methods** | 13a | Describe the processes used to decide which studies were eligible for each synthesis (e.g. tabulating the study intervention characteristics and comparing against the planned groups for each synthesis (item 5)). | 5 |
|  | 13b | Describe any methods required to prepare the data for presentation or synthesis, such as handling of missing summary statistics, or data conversions. | 5 |
|  | 13c | Describe any methods used to tabulate or visually display results of individual studies and syntheses. | 5 |
|  | 13d | Describe any methods used to synthesize results and provide a rationale for the choice(s). If meta-analysis was performed, describe the model(s), method(s) to identify the presence and extent of statistical heterogeneity, and software package(s) used. | 5 |
|  | 13e | Describe any methods used to explore possible causes of heterogeneity among study results (e.g. subgroup analysis, meta-regression). | 5 |
|  | 13f | Describe any sensitivity analyses conducted to assess robustness of the synthesized results. | 5 |
| **Reporting bias assessment** | 14 | Describe any methods used to assess risk of bias due to missing results in a synthesis (arising from reporting biases). | 5 |
| **Certainty assessment** | 15 | Describe any methods used to assess certainty (or confidence) in the body of evidence for an outcome. | 5 |
| **RESULTS** |  |  |  |
| **Study selection** | 16a | Describe the results of the search and selection process, from the number of records identified in the search to the number of studies included in the review, ideally using a flow diagram. | 6 |
|  | 16b | Cite studies that might appear to meet the inclusion criteria, but which were excluded, and explain why they were excluded. | Supplement |
| **Study characteristics** | 17 | Cite each included study and present its characteristics. | 6 -9 |
| **Risk of bias in studies** | 18 | Present assessments of risk of bias for each included study. | 6 |
| **Results of individual studies** | 19 | For all outcomes, present, for each study: (a) summary statistics for each group (where appropriate) and (b) an effect estimate and its precision (e.g. confidence/credible interval), ideally using structured tables or plots. | 6-8 |
| **Results of syntheses** | 20a | For each synthesis, briefly summarise the characteristics and risk of bias among contributing studies. | 6-8 |
|  | 20b | Present results of all statistical syntheses conducted. If meta-analysis was done, present for each the summary estimate and its precision (e.g. confidence/credible interval) and measures of statistical heterogeneity. If comparing groups, describe the direction of the effect. | 6-8 |
|  | 20c | Present results of all investigations of possible causes of heterogeneity among study results. | 6 |
|  | 20d | Present results of all sensitivity analyses conducted to assess the robustness of the synthesized results. | 6 |
| **Reporting biases** | 21 | Present assessments of risk of bias due to missing results (arising from reporting biases) for each synthesis assessed. | 6 |
| **Certainty of evidence** | 22 | Present assessments of certainty (or confidence) in the body of evidence for each outcome assessed. | 6 |
| **DISCUSSION** |  |  |  |
| **Discussion** | 23a | Provide a general interpretation of the results in the context of other evidence. | 10, 13 |
|  | 23b | Discuss any limitations of the evidence included in the review. | 13 |
|  | 23c | Discuss any limitations of the review processes used. | 13 |
|  | 23d | Discuss implications of the results for practice, policy, and future research. | 12-13 |
| **OTHER INFORMATION** |  |  |  |
| **Registration and protocol** | 24a | Provide registration information for the review, including register name and registration number, or state that the review was not registered. | 4 |
|  | 24b | Indicate where the review protocol can be accessed, or state that a protocol was not prepared. | 4 |
|  | 24c | Describe and explain any amendments to information provided at registration or in the protocol. | 4 |
| **Support** | 25 | Describe sources of financial or non-financial support for the review, and the role of the funders or sponsors in the review. | 14 |
| **Competing interests** | 26 | Declare any competing interests of review authors. | 14 |
| **Availability of data, code and other materials** | 27 | Report which of the following are publicly available and where they can be found: template data collection forms; data extracted from included studies; data used for all analyses; analytic code; any other materials used in the review. | 14 |

#####

# PRISMA Abstract Checklist

| **Topic** | **No.** | **Item** | **Reported?** |
| --- | --- | --- | --- |
| **TITLE** |  |  |  |
| **Title** | 1 | Identify the report as a systematic review. | Yes |
| **BACKGROUND** |  |  |  |
| **Objectives** | 2 | Provide an explicit statement of the main objective(s) or question(s) the review addresses. | Yes |
| **METHODS** |  |  |  |
| **Eligibility criteria** | 3 | Specify the inclusion and exclusion criteria for the review. | Yes |
| **Information sources** | 4 | Specify the information sources (e.g. databases, registers) used to identify studies and the date when each was last searched. | Yes |
| **Risk of bias** | 5 | Specify the methods used to assess risk of bias in the included studies. | Yes |
| **Synthesis of results** | 6 | Specify the methods used to present and synthesize results. | Yes |
| **RESULTS** |  |  |  |
| **Included studies** | 7 | Give the total number of included studies and participants and summarise relevant characteristics of studies. | Yes |
| **Synthesis of results** | 8 | Present results for main outcomes, preferably indicating the number of included studies and participants for each. If meta-analysis was done, report the summary estimate and confidence/credible interval. If comparing groups, indicate the direction of the effect (i.e. which group is favoured). | Yes |
| **DISCUSSION** |  |  |  |
| **Limitations of evidence** | 9 | Provide a brief summary of the limitations of the evidence included in the review (e.g. study risk of bias, inconsistency and imprecision). | Yes |
| **Interpretation** | 10 | Provide a general interpretation of the results and important implications. | Yes |
| **OTHER** |  |  |  |
| **Funding** | 11 | Specify the primary source of funding for the review. | Yes |
| **Registration** | 12 | Provide the register name and registration number. | Yes |

*From:* Page MJ, McKenzie JE, Bossuyt PM, Boutron I, Hoffmann TC, Mulrow CD, et al. The PRISMA 2020 statement: an updated guideline for reporting systematic reviews. MetaArXiv. 2020, September 14. DOI: 10.31222/osf.io/v7gm2. For more information, visit: [www.prisma-statement.org](file:///C:\Users\rebelose\Downloads\www.prisma-statement.org)

References

1. Ahrén B, Hirsch IB, Pieber TR, Mathieu C, Gómez-Peralta F, Hansen TK, et al. Efficacy and Safety of Liraglutide Added to Capped Insulin Treatment in Subjects With Type 1 Diabetes: the ADJUNCT TWO Randomized Trial. Diabetes Care [Internet]. 2016;39:1693‐1701. Available from: https://www.cochranelibrary.com/central/doi/10.1002/central/CN-01413998/full

2. Higgins J, Thomas J, Chandler J, Cumpston M, Li T, Page M, et al. Cochrane Handbook for Systematic Reviews of Interventions version 6.4 (updated August 2023) [Internet]. Cochrane. 2023. Available from: www.training.cochrane.org/handbook

3. Brock C, Hansen CS, Karmisholt J, Møller HJ, Juhl A, Farmer AD, et al. Liraglutide treatment reduced interleukin-6 in adults with type 1 diabetes but did not improve established autonomic or polyneuropathy. Br J Clin Pharmacol [Internet]. 2019;85:2512‐2523. Available from: https://www.cochranelibrary.com/central/doi/10.1002/central/CN-01979004/full

4. Wan X, Wang W, Liu J, Tong T. Estimating the sample mean and standard deviation from the sample size, median, range and/or interquartile range. BMC Med Res Methodol. England; 2014;14:135.

5. Dandona P, Ghanim H, Kuhadiya ND, Shah T, Hejna JM, Makdissi A, et al. Liraglutide as an additional treatment to insulin in patients with type 1 diabetes mellitus-A 52-week randomized double-blinded placebo-controlled clinical trial. Diabetes [Internet]. 2018;67 CC-:LB1. Available from: https://www.cochranelibrary.com/central/doi/10.1002/central/CN-01631266/full

6. Dejgaard TF, Frandsen CS, Hansen TS, Almdal T, Urhammer S, Pedersen-Bjergaard U, et al. Efficacy and safety of liraglutide for overweight adult patients with type 1 diabetes and insufficient glycaemic control (Lira-1): a randomised, double-blind, placebo-controlled trial. lancet Diabetes Endocrinol [Internet]. 2016;4:221‐232. Available from: https://www.cochranelibrary.com/central/doi/10.1002/central/CN-01137587/full

7. Dejgaard TF, Frandsen CS, Kielgast U et al. 59-OR: Liraglutide 1, preserved insulin secretion in adults with newly diagnosed type 1):59-OR., diabetes: the NewLira trial. Diabetes. 2019;68:59-OR.

8. Dejgaard TF, Schmidt S, Frandsen CS, Vistisen D, Madsbad S, Andersen HU, et al. Liraglutide reduces hyperglycaemia and body weight in overweight, dysregulated insulin-pump-treated patients with type 1 diabetes: the Lira Pump trial-a randomized, double-blinded, placebo-controlled trial. Diabetes Obes Metab [Internet]. 2020;22:492‐500. Available from: https://www.cochranelibrary.com/central/doi/10.1002/central/CN-02006128/full

9. Dubé MC, D’Amours M, Weisnagel SJ. Beyond glycaemic control: a cross-over, double-blinded, 24-week intervention with liraglutide in type 1 diabetes. Diabetes Obes Metab [Internet]. 2018;20:178‐184. Available from: https://www.cochranelibrary.com/central/doi/10.1002/central/CN-01622136/full

10. Frandsen CS, Dejgaard TF, Holst JJ, Andersen HU, Thorsteinsson B, Madsbad S. Twelve-Week Treatment With Liraglutide as Add-on to Insulin in Normal-Weight Patients With Poorly Controlled Type 1 Diabetes: a Randomized, Placebo-Controlled, Double-Blind Parallel Study. Diabetes Care [Internet]. 2015;38:2250‐2257. Available from: https://www.cochranelibrary.com/central/doi/10.1002/central/CN-01140763/full

11. Ghanim H, Batra M, Green K, Abuaysheh S, Hejna J, Makdissi A, et al. Liraglutide treatment in overweight and obese patients with type 1 diabetes: a 26-week randomized controlled trial; mechanisms of weight loss. Diabetes Obes Metab [Internet]. 2020;22:1742‐1752. Available from: https://www.cochranelibrary.com/central/doi/10.1002/central/CN-02121358/full

12. Abstracts of the 48th EASD (European Association for the Study of Diabetes) annual meeting of the European Association for the Study of Diabetes. October 1-5, 2012. Berlin, Germany. Diabetologia. 2012;55:S300.

13. Herold KC, Reynolds J, Dziura J, Baidal D, Gaglia J, Gitelman SE, et al. Exenatide extended release in patients with type 1 diabetes with and without residual insulin production. Diabetes Obes Metab [Internet]. 2020;22:2045‐2054. Available from: https://www.cochranelibrary.com/central/doi/10.1002/central/CN-02161171/full

14. Johansen NJ, Dejgaard TF, Lund A, Schlüntz C, Frandsen CS, Forman JL, et al. Efficacy and safety of meal-time administration of short-acting exenatide for glycaemic control in type 1 diabetes (MAG1C): a randomised, double-blind, placebo-controlled trial. lancet Diabetes Endocrinol [Internet]. 2020;8:313‐324. Available from: https://www.cochranelibrary.com/central/doi/10.1002/central/CN-02098746/full

15. Kuhadiya ND, Dhindsa S, Ghanim H, Mehta A, Makdissi A, Batra M, et al. Addition of Liraglutide to Insulin in Patients With Type 1 Diabetes: a Randomized Placebo-Controlled Clinical Trial of 12 Weeks. Diabetes Care [Internet]. 2016;39:1027‐1035. Available from: https://www.cochranelibrary.com/central/doi/10.1002/central/CN-01158239/full

16. Hari Kumar K V, Shaikh A, Prusty P. Addition of exenatide or sitagliptin to insulin in new onset type 1 diabetes: a randomized, open label study. Diabetes Res Clin Pract [Internet]. 2013;100:e55‐8. Available from: https://www.cochranelibrary.com/central/doi/10.1002/central/CN-00964892/full

17. Mathieu C, Zinman B, Hemmingsson JU, Woo V, Colman P, Christiansen E, et al. Efficacy and Safety of Liraglutide Added to Insulin Treatment in Type 1 Diabetes: the ADJUNCT ONE Treat-To-Target Randomized Trial. Diabetes Care [Internet]. 2016;39:1702‐1710. Available from: https://www.cochranelibrary.com/central/doi/10.1002/central/CN-01413982/full

18. Navodnik MP, Janež A, Žuran I. The Effect of Additional Treatment with Empagliflozin or Semaglutide on Endothelial Function and Arterial Stiffness in Subjects with Type 1 Diabetes Mellitus-ENDIS Study. Pharmaceutics. Switzerland; 2023;15.

19. Sarkar G, Alattar M, Brown RJ, Quon MJ, Harlan DM, Rother KI. Exenatide treatment for 6 months improves insulin sensitivity in adults with type 1 diabetes. Diabetes Care [Internet]. 2014;37:666‐670. Available from: https://www.cochranelibrary.com/central/doi/10.1002/central/CN-00981863/full

20. Thivolet C, Larger E, Cariou B, Renard E, Hanaire H, Benhamou PY, et al. Dulaglutide and insulin microsecretion in people with type 1 diabetes (DIAMOND-GLP-1): a randomized double-blind placebo-controlled trial. Diabetes Metab [Internet]. 2023;49:101433. Available from: https://www.cochranelibrary.com/central/doi/10.1002/central/CN-02534844/full

21. von Herrath M, Bain SC, Bode B, Clausen JO, Coppieters K, Gaysina L, et al. Anti-interleukin-21 antibody and liraglutide for the preservation of β-cell function in adults with recent-onset type 1 diabetes: a randomised, double-blind, placebo-controlled, phase 2 trial. lancet Diabetes Endocrinol [Internet]. 2021;9:212‐224. Available from: https://www.cochranelibrary.com/central/doi/10.1002/central/CN-02252690/full

22. Zenz S, Regittnig W, Boulgaropoulos B, Augustin T, Brunner M, Korsatko S, et al. Effect of Liraglutide Treatment on Whole-body Glucose Fluxes in C-peptide-Positive Type 1 Diabetes During Hypoglycemia. J Clin Endocrinol Metab [Internet]. 2022;107:e3583‐e3593. Available from: https://www.cochranelibrary.com/central/doi/10.1002/central/CN-02426148/full

23. Jiang LL, Wang SQ, Ding B, Zhu J, Jing T, Ye L, et al. The effects of add-on exenatide to insulin on glycemic variability and hypoglycemia in patients with type 1 diabetes mellitus. J Endocrinol Invest [Internet]. 2018;41:539‐547. Available from: https://www.cochranelibrary.com/central/doi/10.1002/central/CN-01643336/full

24. Kielgast U, Krarup T, Holst JJ, Madsbad S. Four weeks of treatment with liraglutide reduces insulin dose without loss of glycemic control in type 1 diabetic patients with and without residual beta-cell function. Diabetes Care [Internet]. 2011;34:1463‐1468. Available from: https://www.cochranelibrary.com/central/doi/10.1002/central/CN-00799907/full

25. Pieber TR, Deller S, Korsatko S, Jensen L, Christiansen E, Madsen J, et al. Counter-regulatory hormone responses to hypoglycaemia in people with type 1 diabetes after 4 weeks of treatment with liraglutide adjunct to insulin: a randomized, placebo-controlled, double-blind, crossover trial. Diabetes Obes Metab [Internet]. 2015;17:742‐750. Available from: https://www.cochranelibrary.com/central/doi/10.1002/central/CN-01256282/full

26. Ballav C, Dhere A, Kennedy I, Agbaje OF, White S, Franklin R, et al. Lixisenatide in type 1 diabetes: a randomised control trial of the effect of lixisenatide on post-meal glucose excursions and glucagon in type 1 diabetes patients. Endocrinol diabetes Metab [Internet]. 2020;3. Available from: https://www.cochranelibrary.com/central/doi/10.1002/central/CN-02130824/full

27. Van Meijel L, Rooijackers HM, Tack CJ, De Galan BE. Effect of the GLP-1 receptor agonist exenatide on awareness of hypoglycaemia in patients with type 1 diabetes and impaired awareness of hypoglycaemia. Diabetes Technol Ther [Internet]. 2019;21:A132‐A133. Available from: https://www.cochranelibrary.com/central/doi/10.1002/central/CN-01917932/full

28. Dubé MC, D’Amours M, Weisnagel SJ. Effect of liraglutide on food consumption, appetite sensations and eating behaviours in overweight people with type 1 diabetes. Diabetes Obes Metab [Internet]. 2020;22:1417‐1424. Available from: https://www.cochranelibrary.com/central/doi/10.1002/central/CN-02097592/full
