# Supplementary material for: Glucagon-like peptide-1 receptor agonists as add-on therapy to insulin for type 1 diabetes mellitus: a systematic review and meta-analysis
Source: Hormones (Athens). 2025 Aug 4;24(4):1141–51. doi: 10.1007/s42000-025-00704-9 (PMC12678461; doi:10.1007/s42000-025-00704-9)
Supplement: Supplementary file 2 — Supplementary file2 (DOCX 21 KB) [file 42000_2025_704_MOESM2_ESM.docx]

**Supplementary Table 1** – Baseline characteristics of the patients and the studies included

| **Study ID** | **Group** | **Study duration (w)** | **N**  **(F%)** | **Age (yrs)** | **T1D duration (yrs)** | **HbA_1c_ (%)** | **BW (Kg)** | **BMI (Kg/m^2^)** | **C peptide (pmol/L)** |
| --- | --- | --- | --- | --- | --- | --- | --- | --- | --- |
| ***Parallel studies*** |  |  |  |  |  |  |  |  |  |
| Frandsen 2015 | Lira | 12 | 20 (39) | 39.5 ± 2.7 | 18.3 ± 2.0 | 8.8 ± 0.2 | 75.8 ± 2.9 | 24.2 ± 0.6 | 11.2 ± 4.6 |
|  | PBO |  | 20 (28) | 36.1 ± 1.6 | 19.56 ± 1.6 | 8.7 ± 1.4 | 74.9 ± 1.7 | 22.8 ± 0.4 | 7.6 ± 3.7 |
| Brock 2019 | Lira | 26 | 19 (20) | 51 ± 10 | 31 (24-43) | 8.5 (7.5-9.5) | 93 ± 17 | 29 ± 4 | n.m. |
| ’ | PBO |  | 20 (30) | 50 ± 8 | 32 (26-40) | 7.9 (7.3-8.6) | 92 ± 17 | 29 ± 5 | n.m. |
| Mathieu 2016 | Lira 0.6 | 52 | 350 (53.1) | 43.6 ± 12.8 | 20.9 ±12.2 | 8.18 ±0.738 | 86.5 ± 17.3 | 29.5 ± 5.3 | 150^ |
|  | Lira 1.2 |  | 346 (51.7) | 43.9 ± 13.1 | 21.6 ± 12.2 | 8.16 ± 0.779 | 85.4 ± 17.2 | 29.3 ± 5.1 | 130^ |
|  | Lira 1.8 |  | 346 (52.3) | 43.7 ± 13.3 | 21.5 ±12.6 | 8.14 ± 0.740 | 86.3 ± 17.3 | 29.5 ± 5.2 | 111^ |
|  | PBO |  | 347 (51.9) | 43.4 ± 12.6 | 21.6 ± 11.8 | 8.15 ± 0.728 | 86.4 ± 17.8 | 29.8 ± 5.6 | 130^ |
| Ahren 2016 | Lira 0.6 | 26 | 211 (56) | 43.9 (19;87) | 21.0 (1; 58) | 8.09 | 83.1 | 28.9 | 52 |
|  | Lira 1.2 |  | 209 (51) | 42.8 (18;73) | 21.1 (1; 52) | 8.07 | 84.7 | 28.8 | 31 |
|  | Lira 1.8 |  | 206 (55) | 43.2 (18;75) | 21.4 (1; 53) | 8.04 | 83.6 | 28.9 | 37 |
|  | PBO |  | 206 (54) | 42.7 (18;70) | 20.7 (1; 54) | 8.12 | 84.2 | 28.9 | 33 |
| Dejgaard 2020 | Lira | 26 | 22 (68) | 50± 14 | 21 (15-34) | 8.2 ± 0.5 | 85 ± 10 | 30 ± 2 | 9 (7-18)# |
|  | PBO |  | 22 (68) | 43 ± 12 | 20 (15-35) | 8.1 ± 0.5 | 88 ± 14 | 29 ± 3 | 9 (6-12)# |
| Jiang 2018 | Exe | 4 | 15 (nm) | 40.3 ± 7.4 | 11.0 ± 10.0 | 9.3 ± 2.8 | 57.7 ± 79 | 21.6 ± 3.2 | n.m. |
|  | PBO |  | 15 (nm) | 38.7 ± 7.2 | 9.1 ± 5.4 | 8.8 ± 2.6 | 59.5 ± 8.2 | 21.5 ± 2.4 | n.m. |
| Navodnik 2023 | Sema | 12 | 30 (36.7) | 48.5 ± 9.6 | 21.9 ± 11.2 | 7.42 ± 0.80 | 84.6 ± 16.4 | 28.2 ± 4.8 | n.m. |
|  | PBO |  | 29 (41.4) | 47.0 ± 12 | 18.8 ± 11.9 | 7.04 ± 0.9 | 82.6 ± 11.7 | 26.9 ± 3.0 | n.m. |
| Johansen 2020 | Exe | 26 | 52 (25) | 50.1 ± 14.2 | 21.2 ± 11.3 | 8.3 ± 0.80 | 89.7 ± 14.4 | 29.0 ± 4.8 | 24.6 (53.2) |
|  | PBO |  | 53 (30) | 50.4 ± 14 | 21.0 ± 12.9 | 8.2 ± 0.6 | 85.8 ± 14.3 | 27.7 ± 4.1 | 23.2 (64.2) |
| Dejgaard 2016 | Lira | 24 | 50 (40) | 47 ± 13 | 20 ± 12 | 8.7 ± 0.7 | 93.4 ± 14.2 | 30.3 ± 3.5 | 11 ± 72 |
|  | PBO |  | 50 (30) | 49 ± 12 | 25 ± 12 | 8.7 ± 0.7 | 94 ± 12.5 | 29.8 ± 3.1 | 22 ± 30 |
| Ghanim 2020 | Lira | 26 | 37 (65) | 47±12.17 | 19 ± 12.2 | 7.96 ± 1.16 | 94.2 ± 18.9 | 33.3 ± 7.3 | n.m. |
|  | PBO |  | 27 (59) | 45±15.59 | 18 ± 15.6 | 7.79 ± 0.94 | 83.3 ± 17.7 | 29.5 ± 6.8 | n.m. |
| Dejgaard 2019 | Lira |  | 65 (nm) | 27 ± 5 | 4.4± 1.2^§^ | 8.3 (7.8-8.6) | n.m. | 24 (22 to 25) | 359±226** |
|  | PBO |  |  |  |  | 8.6 (8.1-9.0) |  | 24 (22 to 24) |  |
| Von Herrath 2021 | Lira | 54 | 76 (33) | 28 ± 7.1 | 10.8 ± 4.8^§^ | 7.2 ± 1.5 | 74 ± 13.8 | 24.2 ± 3.8 | 240 (70.4%) |
|  | PBO |  | 77 (36) | 29 ± 7 | 10.2 ± 4.7^§^ | 7.3 ± 1.3 | 72.8 ± 19.8 | 24.0 ± 5.0 | 230 (83.7%) |
| Kuhadiya 2016 | Lira 0.6 | 12 | 14 (50) | 45 ± 4^&^ | 25 ± 2 | 7.46 ± 0.19 | 80±4 | 26 ± 3 | n.m. |
|  | Lira 1.2 |  | 16 (50) | 42 ± 3^&^ | 21 ± 3 | 7.84 ± 0.17 | 96±4 | 33 ± 2 | n.m. |
|  | Lira 1.8 |  | 16 (28) | 42 ± 3^&^ | 20 ± 3 | 7.41±0.15 | 83±4 | 28 ± 4 | n.m. |
|  | PBO |  | 17 (71) | 50 ± 3^&^ | 30 ± 3 | 7.69±0.17 | 80±6 | 28 ± 2 | n.m. |
| Kielgast 2011 | Lira | 4 | 9 (0) | 35.7±2.2 | 17.3±2.5 | 7.5 ± 0.2 | n.m. | 24.6 ± 0.7 | n.m. |
|  | PBO |  | 10 (10) | 32.9 ± 1.7 | 23.1±1.6 | 7.1±0.3 | n.m. | 23.1 ± 0.6 | n.m. |
| Herold 2020 | Exe | 24 | 40 (72) | 38.6 ± 12.1 | 22.1 ± 12.0 | 7.4 ± 0.8 | 83.7 ± 21.7 | 29.3 ± 6.4 | n.m. |
|  | PBO |  | 39 (64) | 33.5 ± 11.4 | 17.1 ± 9.8 | 7.8 ± 0.8 | 84.1 ± 22.6 | 29.4 ± 6.3 | n.m. |
| Thivolet 2023 | Dula | 24 | 8 (nm) | 43.2 [36.1;50.4] | 12.3 [9.3;14.9] | 8.35 [7.92;8.85] | 79.6 [69;90.8] | 27.8 [24.5;29.9] | 60 [29.3;90.7] |
|  | PBO |  | 10 (nm) | 38.8 [32.4;45.2] | 10.6 [7.9;13.3] | 7.87 [7.44;8.28] | 75.8[66.6;86.1] | 25.2 [22.7;27.3] | 79 [41.5;117] |
| Kumar 2013 | EXE | 52 | 6 (n.m.) | 28.8 ± 7.6 | 29.6 ± 8.8* | 9.7 ± 0.8 | 56.2±3.4 | 21.5 ± 1.7 | 159 ± 83 |
|  | PBO |  | 6 (n.m.) | 27.5 ± 4.9 | 35.9 ± 9.7* | 9.9 ± 0.9 | 58.3±7.3 | 21.7 ± 3.4 | 132 ± 66 |
| Dandona 2018 | Lira | 52 | 20 (n.m.) | 46.7 ± 1.9 | 22.3 ± 1.7 | 7.82 ± 0.16 | n.m. | 28.9 ± 1.4 | n.m. |
|  | PBO |  | 20 (n.m.) |  |  |  |  |  | n.m. |
| Hamamoto 2012 | Lira 0.9 | 52 | 10 (nm) | 48.5±12.1 | n.m. | 7.6 ± 1.4 |  | 22.8 ± 1.8 | n.m. |
|  | PBO |  |  |  |  | 7.7 ± 0.7 |  |  | n.m. |
| ***Crossover studies*** |  |  |  |  |  |  |  |  |  |
| Pieber 2015 | Lira 0.6 | 4 | 15 (40) | 38.9 ± 11.3 | 17.7 ± 9.2 | 7.84 ± 0.88 | 75.4±14.0 | 24.1 ± 2.6 | n.m. |
|  | Lira 1.2 |  | 14 (21.4) | 34.6 ± 12.5 | 18.5 ± 10.3 | 7.49 ± 0.71 | 72.5±8.9 | 23.2 ± 1.9 | n.m. |
|  | Lira 1.8 |  | 16 (43.8) | 30.4 ± 9.0 | 13.9 ± 8.9 | 7.56 ± 0.68 | 74.2±11.2 | 24.4±2.4 | n.m. |
| Zenz 2022 | Lira | 12 | 14 (50) | 33.6 ± 12.1 | 3.4 ± 2 | 6.9 (5.7, 8.5) | 70.7 (53.1, 95.3) | 23.0 (19.0, 27.0) | 190 (50, 580) |
|  | PBO |  | 14 (50) | 33.6 ± 12.1 | 3.4 ± 2 | 6.7 (5.5, 8.8) | 71.1 (50.9, 94.7) | 22.8 (18.9, 26.8) | 210 (100, 550) |
| Ballav 2020 | Lixisenatide | 4 | 27 (48.1) | 44 ± 2.5 | 18.6 ± 14.2 | 7.9 ± 0.5 | 78.8 ± 11.1 | 27 ± 3.5 | 30 ± 40 |
| Dubé 2018 | Lira | 24 | 15 (53) | 35.8 ± 1.7 | 20.3 ± 2.2 | 7.4 ± 2.2 | 89.0 ± 3.8 | 30.5 ± 0.9 | n.m. |
| Van Meijel 2019 | Exe | 6 | 10 (60) | 38.5 ± 14 | 21.7 ± 13.5 | 7.2 ± 0.4 | 78.1 (68.5-106.3) | 25.4 (23.7-31.7) | n.m. |
| Sarkar 2014 | Exe | 26 | 13 (54) | 37.3 ± 10.7 | 20.5 ± 11.8 | 7.0 ± 0.8 | n.m. | 26.1 ± 3.5 | n.m. |

*T1D duration in this study is in days. § T1D duration in this study is in weeks

#stimulated C-peptide

Dejgaard et al. 2019 reported data as median or mean (95% CI).

In Kuhadiya et al.2016 ^&^ data are mean and SE.

** In Dejgaard 2019, C-peptide levels refer to both groups.

^In Mathieu et al. C-peptide levels refer to a subset of patients, who were C-peptide positive.
